# Supplementary material for: BAF-1–VRK-1 mediated release of meiotic chromosomes from the nuclear periphery is important for genome integrity
Source: Nat Commun. 2025 Nov 25;16:10446. doi: 10.1038/s41467-025-65420-9 (PMC12647756; doi:10.1038/s41467-025-65420-9)
Supplement: Supplementary file 1 — Supplementary Information [file 41467_2025_65420_MOESM1_ESM.pdf]

BAF-1–VRK-1 mediated release of meiotic chromosomes from the nuclear periphery is important for genome integrity

Paouneskou *et. al*

Supplementary Information

Supplementary Table 1: Viability and brood size of strains generated

**Genotype**[1]	**% Average Viability ± SD**	**Average Brood size ± SD**
N2	99.8% ± 0.3	216.4 ± 4
vrk-1(syb2608 vrk-1::AID::ha); tir-1::mRuby (ethanol)	98.1% ± 2.5	242.9 ± 24.7
vrk-1(syb2608 vrk-1::AID::ha); tir-1::mRuby (auxin)	0.0% ± 0.0	211.4 ± 82.4
flag::baf-1	99.7% ± 0.4	198.3 ± 25
flag::baf-1^{T3A}	99% ± 1.1	231 ± 59.2
flag::baf-1^{S4A}	0.0% ± 0.0	31.8 ± 34.6

Supplementary Table 2: % Pairing per germline zone for *vrk-1::AID::ha* germlines

%Mean ± %SD	Zone 1	Zone 2	Zone 3	Zone 4	Zone 5	Zone 6	Zone 7
Pairing 18h ethanol	18.3% ± 5.4%	28.1% ± 12.8%	68.6% ± 17.8%	88.5% ± 3.3%	89.2% ± 5.3%	95.7% ± 2.1%	89.9% ± 1.0%
Pairing 18h auxin	16.0% ± 4.7%	22.7% ± 10.2%	47.4% ± 14.0%	81.6% ± 6.6%	83.2% ± 5.0%	90.5% ± 5.9%	85.6% ± 6.2%
P- value	*P*=0.3115	*P*=0.3877	*P*=0.0002	*P*=0.0993	*P*=0.0937	*P*=0.0409	*P*=0.388
Significance	ns	ns	***	ns	ns	*	ns

All P-values have been calculated using the Two-sided Fischer's exact test.

[1] Italics designates the gene and allele names.

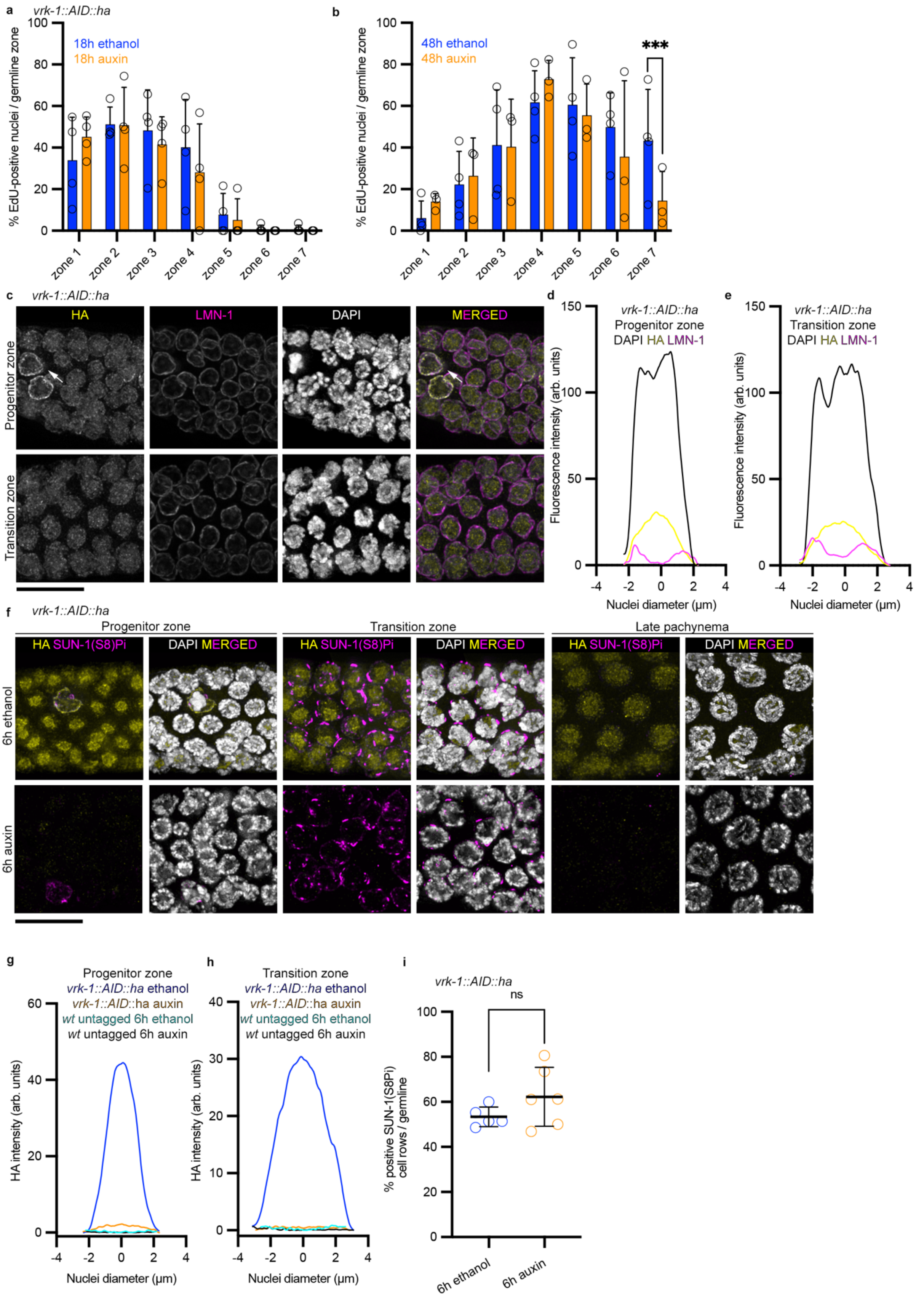

Supplementary Fig. 1. VRK-1 resides within the nuclear envelope and can be efficiently depleted

a, Percentage of EdU-labeled nuclei per germline zone after 18h of ethanol or auxin treatment. The mean ± SD per zone, the n numbers and the exact *P*-values are included in the Source File. Two-sided

Fisher's exact test was used to assess statistical significance. EdU = 5-ethynyl-2′-deoxyuridine. **b,** Percentage of EdU-labeled nuclei per germline zone after 48h of ethanol or auxin treatment. The mean ± SD per zone, the n numbers and the exact *P*-values are included in the Source File. Two-sided Fisher's exact test was used to assess statistical significance. **c,** Monochromatic insets of the indicated meiotic stages in *vrk-1::AID::ha* germlines immunostained against HA (yellow in merged), LMN-1 (magenta in merged) and co-stained with DAPI (white). Scale bar: 10 µm. HA = hemmaglutinin, LMN-1 = lamin 1 (two independent experiments). **d, e,** Average line profile analysis of nuclear HA, LMN-1 and DAPI signal intensities in the progenitor zone (n= 26 nuclei) (**d**) and transition zone (n= 32 nuclei) (**e**). **f,** Insets of the indicated meiotic stages in *vrk-1::AID::ha* germlines immunostained against HA (yellow) and co-stained with DAPI (white) after 6h of ethanol or auxin treatment. Scale bar: 10 µm (three independent experiments). **g, h,** Average line profile analysis of nuclear HA signal intensity for the indicated conditions and genotypes in the progenitor zone (*vrk-1::AID::ha*: ethanol n=37 nuclei, auxin n=44 nuclei; N2 (wild type untagged): ethanol n=34 nuclei, auxin n=37 nuclei) (**g**) and transition zone (*vrk-1::AID::ha*: ethanol n=41 nuclei, auxin: n=47 nuclei; N2 (wild type untagged): ethanol n=37 nuclei, auxin n=34 nuclei) (**h**). **i,** Percentage of cell rows positive for SUN-1 (S8Pi) per *vrk-1::AID::ha* germline for the indicated conditions. % Mean ± SD, 6h ethanol (n=5 germlines): 53.5% ± 4.4% vs 6h auxin (n=6 germlines): 62.2% ± 13.1%, *P*= 0.0953 (Two-sided Fisher's exact test).

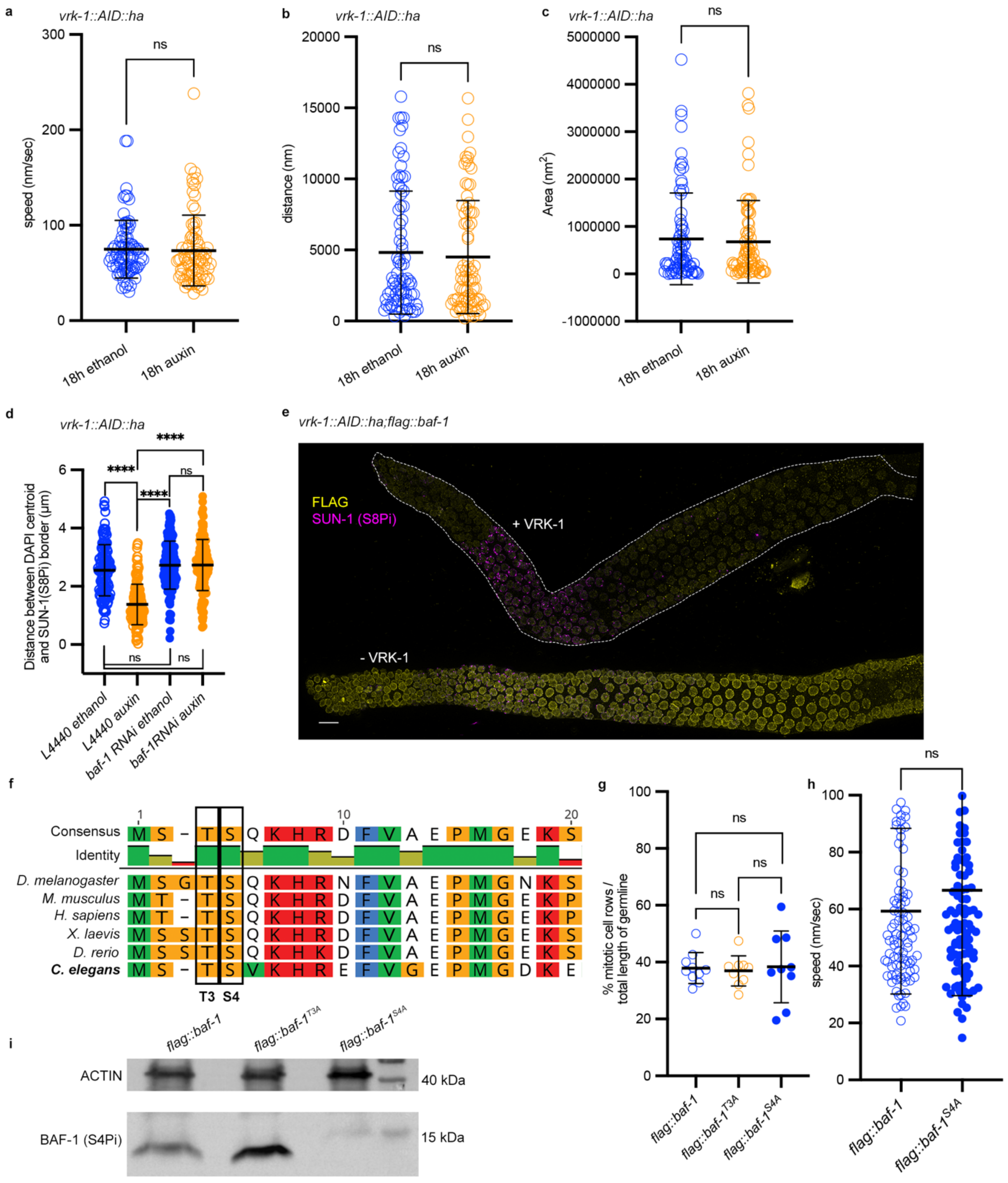

Supplementary Fig. 2. VRK-1 directly regulates BAF-1 but not chromosome dynamics

a, Speed of individual SUN-1::mRuby aggregates in transition zone nuclei of worms after 18 hours of ethanol treatment (mean ± SD: 74.9 ± 30.2 nm/sec, n =77 aggregates) or auxin treatment (mean ± SD: 73. 5 ± 37.2 nm/sec, n=78 aggregates), *P*=0.2337. **b,** Distance travelled by the individual SUN-1::mRuby aggregates in transition zone nuclei of worms after 18 hours of ethanol treatment (mean ± SD: 4,821.8 ± 4,325.2 nm, n=77 aggregates) or auxin treatment (mean ± SD: 4,500.5 ± 3,974.9 nm, n=78 aggregates), *P*=0.6782. **c,** Area covered by the individual SUN-1::mRuby aggregates within transition

zone nuclei of worms after 18 hours of ethanol treatment. Mean ± SD: 739,162.9 ± 968,309.9 nm^2, n =75 aggregates) or auxin treatment (mean ± SD: 678,545.6 ± 868,683.8 nm^2, n=73 aggregates), *P*=0.8867. The two-sided Mann–Whitney test was used to assess statistical significance for **a**, **b**, **c**. **d,** Quantification of the distance between the DAPI centroid and the oocyte border (marked by SUN-1 (S8Pi) for the indicated conditions. The mean ± SD, n number and exact *P*- values are included in the Source table. *P*-values were calculated with the two-sided Kruskal-Wallis test and corrected for multiple comparisons with the Dunn's method. **e,** *vrk-1::AID::ha;flag::baf-1* germlines immunostained for FLAG (yellow) and PiSer8 SUN-1 (magenta) before and after VRK-1 depletion (three independent experiments). **f,** Multiple alignment of BAF-1 for the indicated species. Alignment was performed using Geneious Alignment and Blosum62 as a cost matrix. **g,** Percentage of mitotic cell rows to total length of germline in the indicated genotypes. The mean ± SD per zone, and the exact *P*-values are included in the Source File. Two-sided Fisher's exact test was used to assess statistical significance (n=9 germlines per genotype). **h,** Speed of individual SUN-1::mRuby aggregates in the transition zone nuclei of the indicated genotypes: Mean ± SD: *flag::baf-1*, 59.3 ± 29.1 nm/sec, n=93 aggregates); and *flag::baf-1*^{S4A} (66.6 ± 37 nm/sec, n=105 aggregates); *P*=0.1222, Two-sided Mann–Whitney test. **i,** Western blot of whole worm extracts with the specified antibodies for the indicated genotypes (one independent experiment).

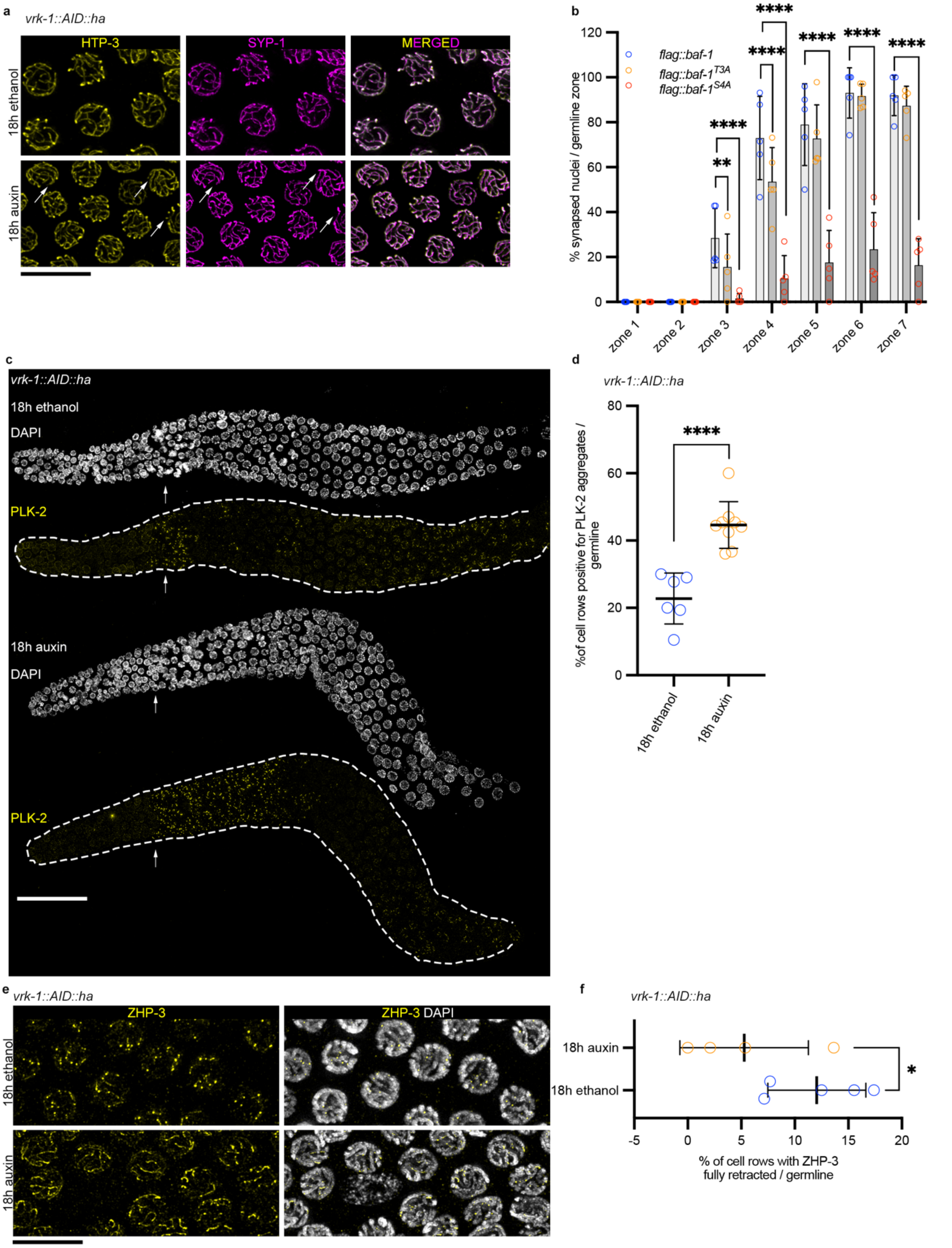

Supplementary Fig. 3. VRK-1 depletion and abrogation of BAF-1 Ser4 phosphorylation impair efficient synapsis, and absence of VRK-1 delays the retraction of PLK-2 and ZHP-3 to crossover sites
a, Late pachynema insets from *vrk-1::AID::ha* germlines immunostained for HTP-3 (yellow) and SYP-1 (magenta) after 18h of ethanol or auxin treatment. Arrows indicate the unsynapsed regions stained only for HTP-3. Scale bar: 10 µm; (3 independent experiments). **b,** Percentage of fully synapsed nuclei

per germline zone for the indicated genotypes (n=5 for all genotypes). The mean ± SD per zone and the exact *P*-values are included in the Source File. Two-sided Fisher's exact test was used to assess statistical significance. **c,** *vrk-1::AID::ha* germlines stained for DAPI (white) and immunostained against PLK-2 (yellow) after 18h of ethanol or auxin treatment. Scale bar: 10 µm; (three independent experiments). **d,** Percentage of cell rows positive for PLK-2 aggregation in the *vrk-1::AID::ha* germlines in ethanol and auxin. Mean ± SD: ethanol (n=6 germlines): 22.8% ± 7.6% vs auxin (n=9 germlines): 44.6% ± 6.9%, *P*<0.0001, Two-sided Fisher's exact test **e.** Late pachynema insets from *vrk-1::AID::ha* germlines immunostained for ZHP-3 (yellow) and co-stained with DAPI (white) after 18h of ethanol or auxin treatment (one independent experiment). Scale bar: 10 µm. **f,** Percentage of cell rows in *vrk-1::AID::ha* germlines with fully retracted ZHP-3 after ethanol or auxin treatment. Mean ± SD: ethanol (n=5 germlines): 12.1% ± 4.6% vs auxin (n=4 germlines): 5.3% ± 6%, *P*=0.0281, Two-sided Fisher's exact test.

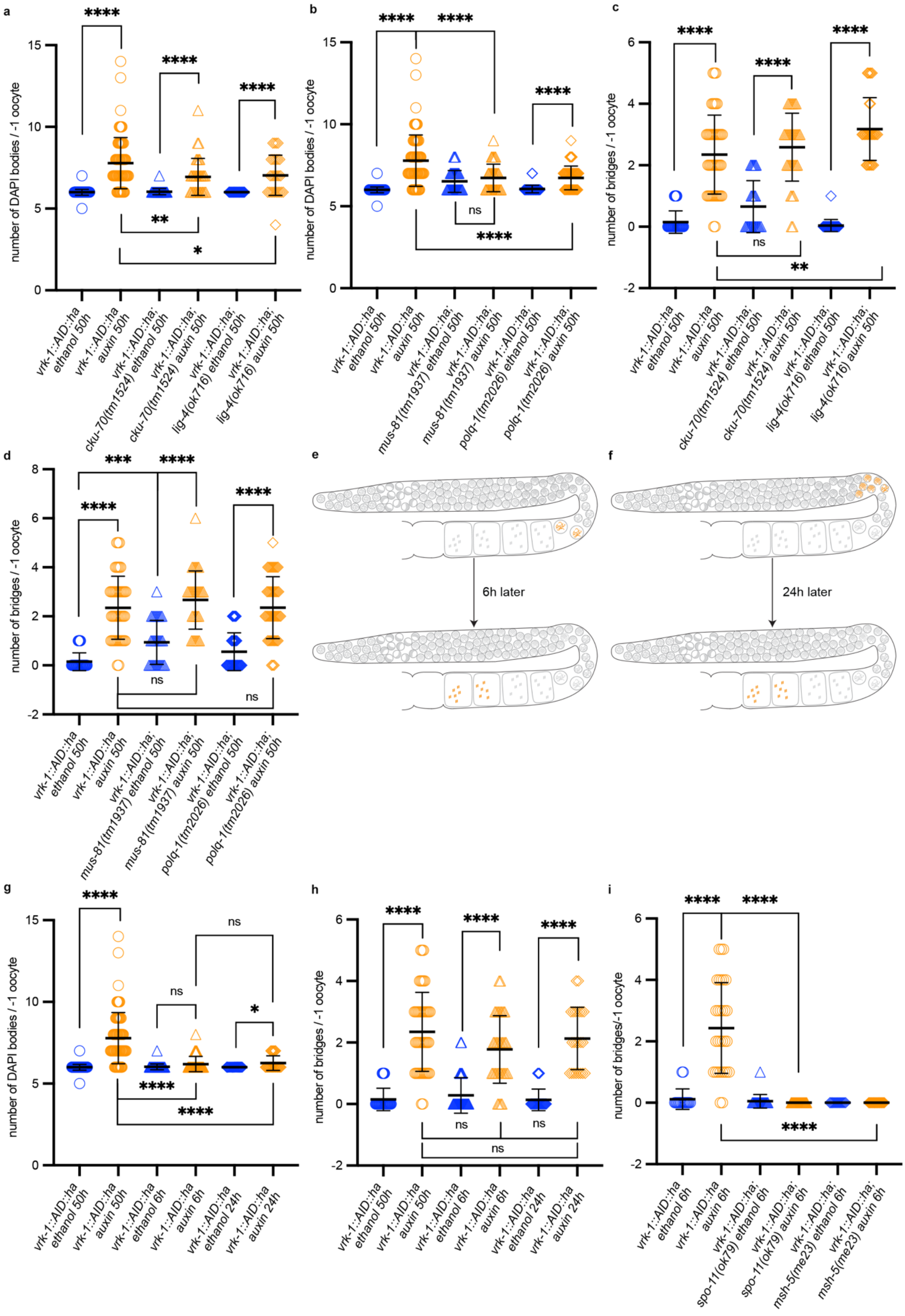
a
number of DAPI bodies / -1 oocyte
vrk-1::AID::ha ethanol 50h
vrk-1::AID::ha auxin 50h
vrk-1::AID::ha; cku-70(tm1524) ethanol 50h
vrk-1::AID::ha; cku-70(tm1524) auxin 50h
vrk-1::AID::ha; lig-4(ok716) ethanol 50h
vrk-1::AID::ha; lig-4(ok716) auxin 50h
b
number of DAPI bodies / -1 oocyte
vrk-1::AID::ha ethanol 50h
vrk-1::AID::ha auxin 50h
vrk-1::AID::ha; mus-81(tm1937) ethanol 50h
vrk-1::AID::ha; mus-81(tm1937) auxin 50h
vrk-1::AID::ha; polq-1(tm2026) ethanol 50h
vrk-1::AID::ha; polq-1(tm2026) auxin 50h
c
number of bridges / -1 oocyte
vrk-1::AID::ha ethanol 50h
vrk-1::AID::ha auxin 50h
vrk-1::AID::ha; cku-70(tm1524) ethanol 50h
vrk-1::AID::ha; cku-70(tm1524) auxin 50h
vrk-1::AID::ha; lig-4(ok716) ethanol 50h
vrk-1::AID::ha; lig-4(ok716) auxin 50h
d
number of bridges / -1 oocyte
vrk-1::AID::ha ethanol 50h
vrk-1::AID::ha auxin 50h
vrk-1::AID::ha; mus-81(tm1937) ethanol 50h
vrk-1::AID::ha; mus-81(tm1937) auxin 50h
vrk-1::AID::ha; polq-1(tm2026) ethanol 50h
vrk-1::AID::ha; polq-1(tm2026) auxin 50h
e
6h later
f
24h later
g
number of DAPI bodies / -1 oocyte
vrk-1::AID::ha ethanol 50h
vrk-1::AID::ha auxin 50h
vrk-1::AID::ha ethanol 6h
vrk-1::AID::ha auxin 6h
vrk-1::AID::ha ethanol 24h
vrk-1::AID::ha auxin 24h
h
number of bridges / -1 oocyte
vrk-1::AID::ha ethanol 50h
vrk-1::AID::ha auxin 50h
vrk-1::AID::ha ethanol 6h
vrk-1::AID::ha auxin 6h
vrk-1::AID::ha ethanol 24h
vrk-1::AID::ha auxin 24h
i
number of bridges/-1 oocyte
vrk-1::AID::ha ethanol 6h
vrk-1::AID::ha auxin 6h
vrk-1::AID::ha; spo-11(ok79) ethanol 6h
vrk-1::AID::ha; spo-11(ok79) auxin 6h
vrk-1::AID::ha; msh-5(me23) ethanol 6h
vrk-1::AID::ha; msh-5(me23) auxin 6h

Supplementary Fig. 4. Alternative DSB repair and timing of DSB repair underlies aberrant chromosome structures in VRK-1 depleted oocytes

a, b, Average number of DAPI bodies per -1 oocyte in the indicated genotypes after 50h of ethanol or auxin treatment. The mean ± SD per zone, n numbers and the exact *P*-values are included in the Source File. Two-sided Mann–Whitney test was used to assess statistical significance in both. **c, d,** Average number of chromosome bridges per -1 oocyte for the indicated genotypes after 50h of ethanol or auxin treatment. The mean ± SD per zone, n numbers and the exact *P*-values are included in the Source File. Two-sided Mann–Whitney test was used to assess statistical significance in both. **e, f,** Schematic representation of the migration of nuclei at 6h (**e**) and 24h (**f**) after the start of VRK-1 depletion. **g,** Average number of DAPI bodies per -1 oocyte for the indicated conditions. The mean ± SD per zone, n numbers and the exact *P*-values are included in the Source File. Two-sided Mann–Whitney test was used to assess statistical significance. **h,** Average number of chromosome bridges per -1 oocyte for the indicated conditions. The mean ± SD per zone, n numbers and the exact *P*-values are included in the Source File. Two-sided Mann–Whitney test was used to assess statistical significance. **i,** Average number of chromosome bridges per -1 oocyte for the indicated genotypes after 6h of ethanol or auxin treatment. The mean ± SD per zone, n numbers and the exact *P*-values are included in the Source File. Two-sided Mann–Whitney test was used to assess statistical significance.

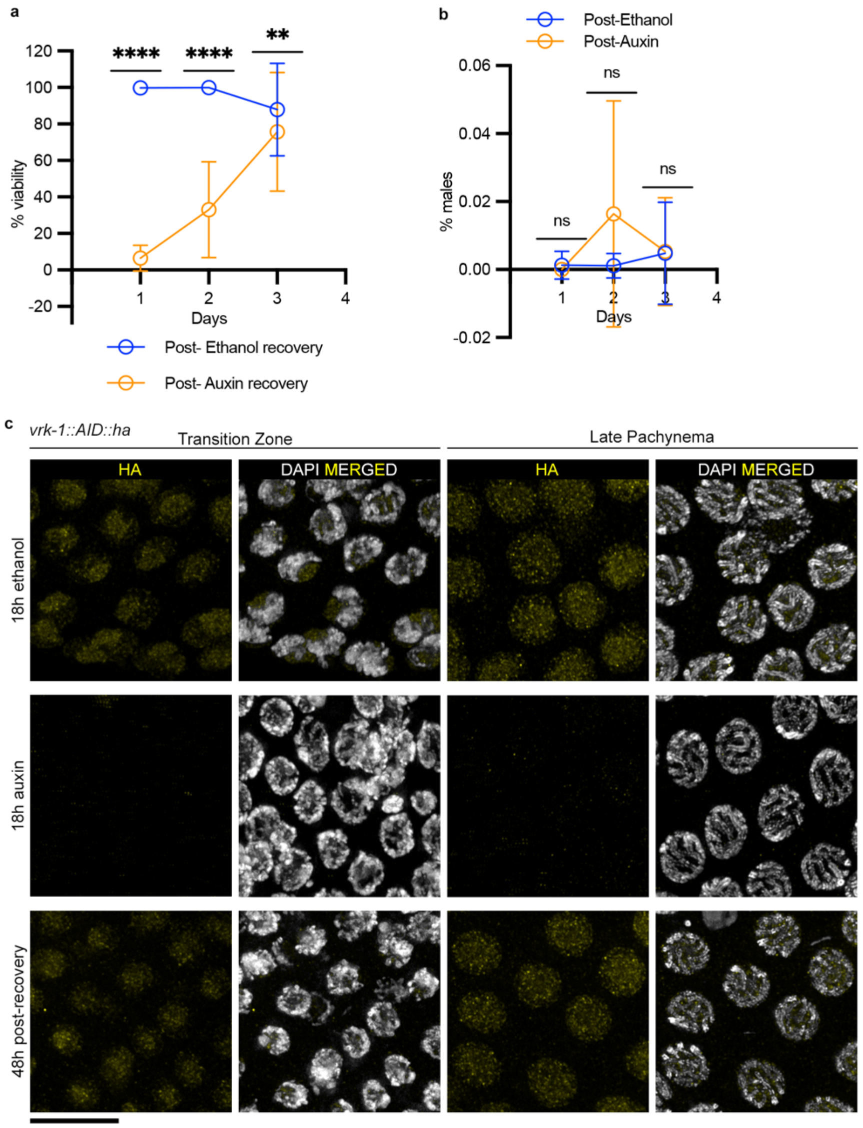

Supplementary Fig. 5. Transient VRK-1 depletion during leptonema–zygonema and early pachynema allows for the generation of viable offspring

a, Percentage viability of offspring up to 3 days after VRK-1 depletion for 18h. Mean ± SD: *vrk-1::AID::ha*, day 1 ethanol (n=10 worms): 99.7% ± 0.5% vs auxin (n=10 worms): 6.6% ± 7.1%, *P*<0.0001; day 2, post ethanol: 99.8% ± 0.5% vs auxin: 33.1% ± 26.2%, *P*<0.0001; and day 3, ethanol: 87.9% ± 25.4% vs auxin: 75.7% ± 32.5%, *P*=0.0021. Two-sided Fisher's exact test was used to assess statistical significance. **b,** Percentage of males of offspring up to 3 days after VRK-1 depletion for 18h. Mean ± SD: *vrk-1::AID::ha*, day 1 ethanol (n=10 worms): 0.0% ± 0.0% vs auxin (n=10 worms): 0.0% ± 0.0%, *P*>0.9999; day 2, post ethanol: 0.0% ± 0.0% vs auxin: 0.01% ± 0.03%, *P*= 0.2272; and day 3, ethanol: 0.00% ± 0.01% vs auxin: 0.00% ± 0.01%, *P*>0.9999. Two-sided Fisher's exact test was used to assess statistical significance. **c,** Insets from *vrk-1::AID::ha* germlines immunostained for HA (yellow) and co-stained with DAPI (white) after 18h of ethanol or auxin treatment and 48h of recovery for the indicated meiotic stages. Scale bar: 10 µm; (one independent experiment). HA = hemmaglutinin

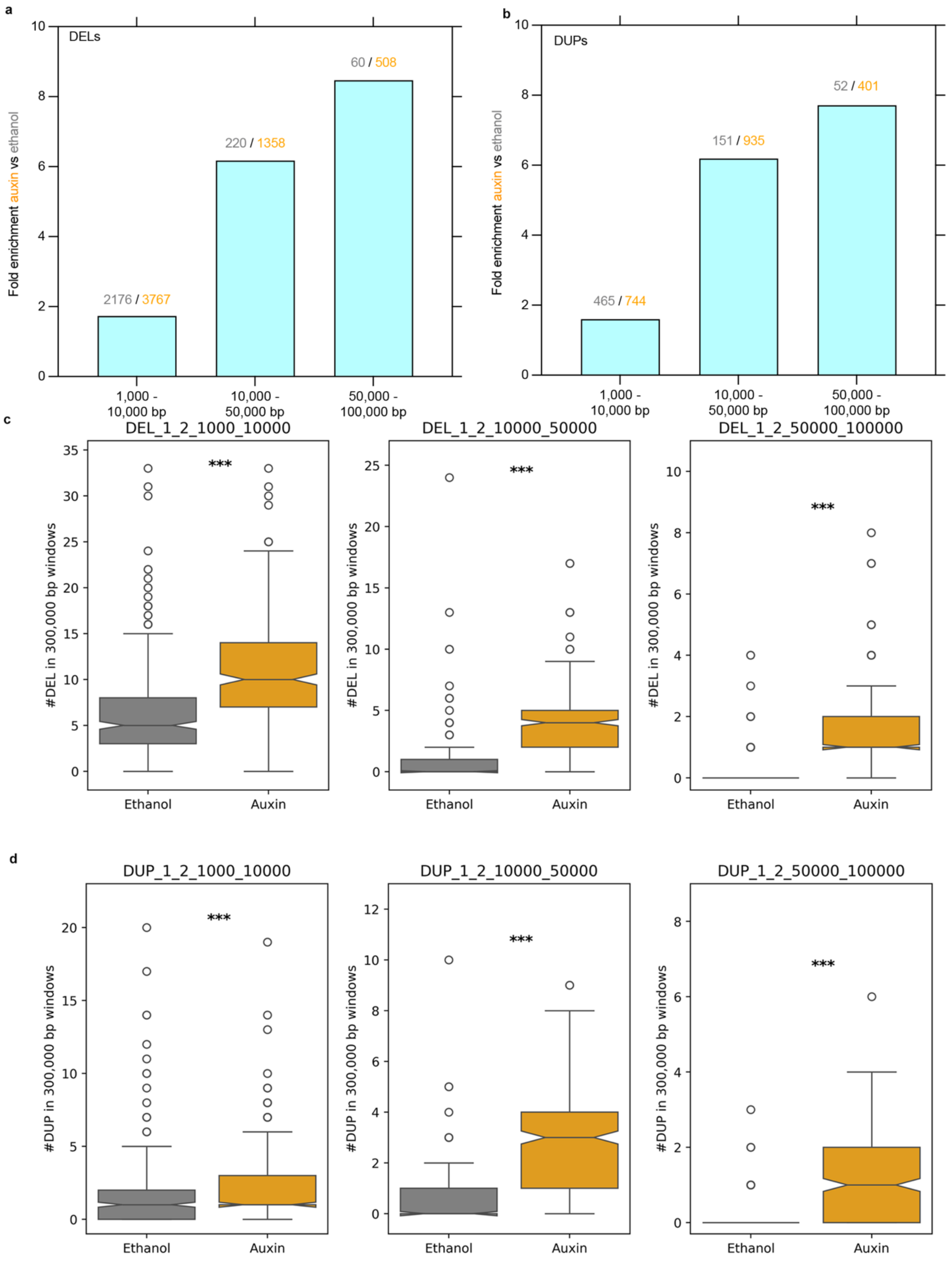

Supplementary Fig. 6. DELs and DUPs are significantly increased upon VRK-1 depletion

a, b, Fold enrichment of DELs (**a**) and DUPs (**b**) for the different size ranges (1,000–10,000, 10,000–50,000, and 50,000–100,000 bp). Raw numbers for ethanol (gray) and auxin (orange) treatments are shown above each column. **c, d,** Numbers of DELs (**c**) and DUPs (**d**) detected for different size classes

after ethanol (gray) or auxin (orange) treatment in non-overlapping windows of 300,000 bp across the genome.

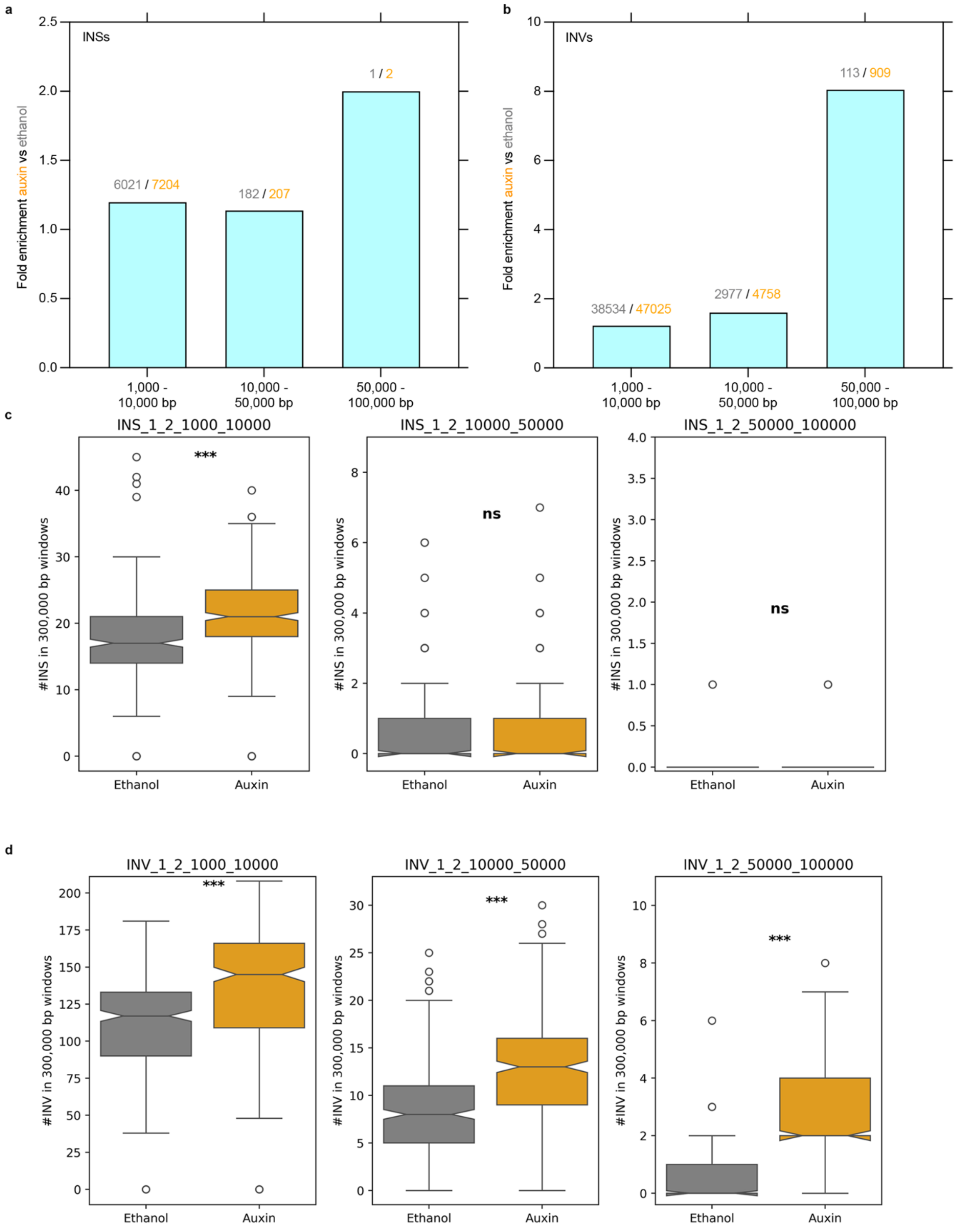

Supplementary Fig. 7. INSs and INVs are overall not significantly increased upon VRK-1 depletion

a, b, Fold enrichment of INSs (**a**) and INVs (**b**) for the different size ranges (1,000–10,000, 10,000–50,000, and 50,000–100,000 bp). The raw numbers for ethanol (gray) and auxin (orange) treatments are shown above each column. **c, d,** Numbers of INS (**c**) and INVs (**d**) detected for different size classes

after ethanol (gray) or auxin (orange) treatment in non-overlapping windows of 300,000 bp across the genome.

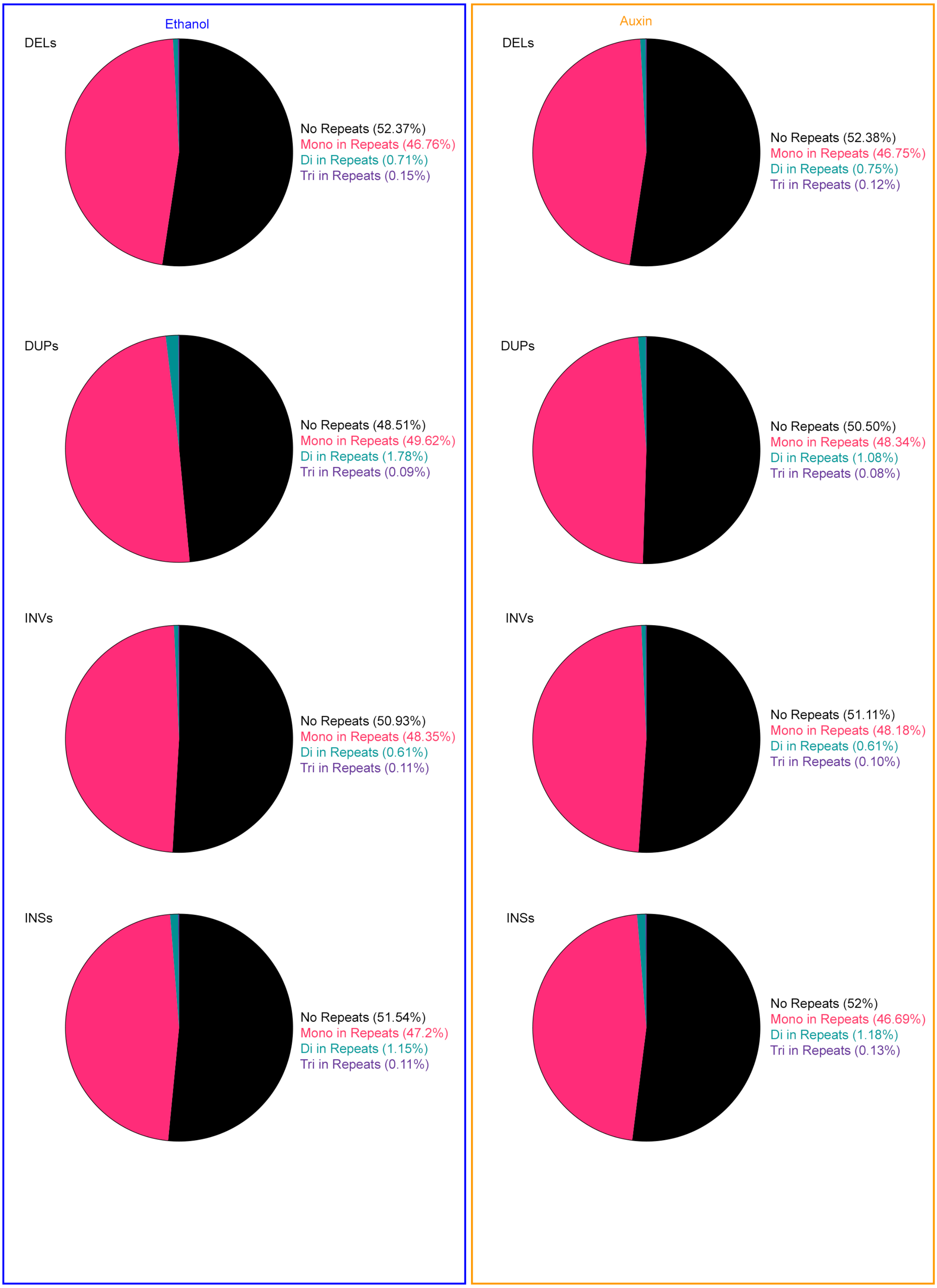

Supplementary Fig. 8. Enrichment of short tandem repeats in flanking regions categorized by deletions (DELs), duplications (DUPs), inversions (INVs), or insertions (INSs) after ethanol (left, blue

frame) or auxin (right, orange frame) treatment. The analysis was restricted to structural variants supported by 1 or 2 sequencing reads that met a minimum length threshold of 1,000 bp.

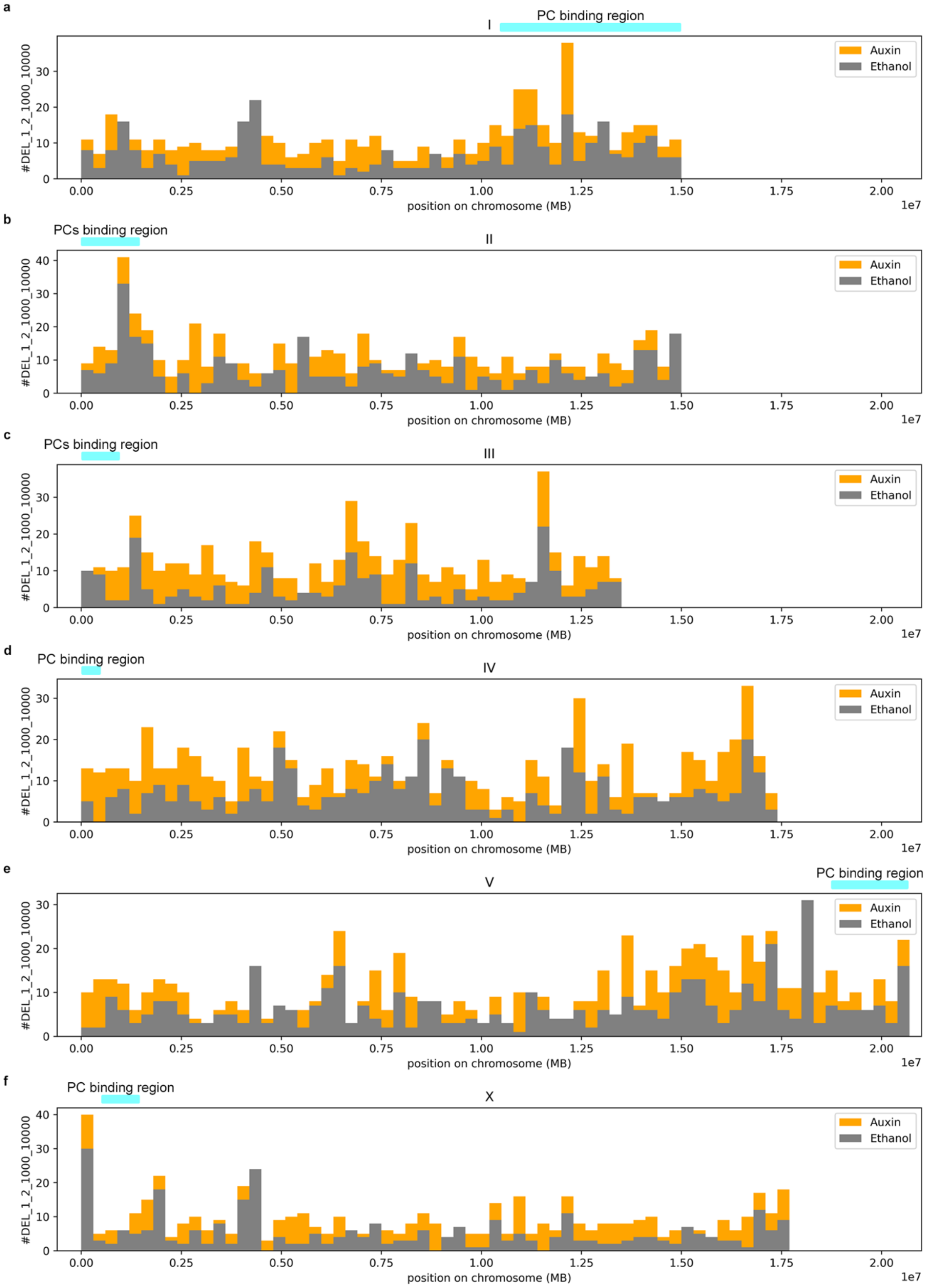

Supplementary Figs 9–11. Distribution of deletions along *C. elegans* chromosomes (bin size 300,000 bp) Supplementary Fig. 9. Number of DELs of size 1,000–10,000 bp supported by 1 and 2 reads along linkage groups I (**a**), II (**b**), III (**c**), IV (**d**), V (**e**), and X (**f**) in samples from ethanol-treated (gray) and auxin-treated (orange) worms. The PC binding region is indicated (cyan). PC = pairing center.

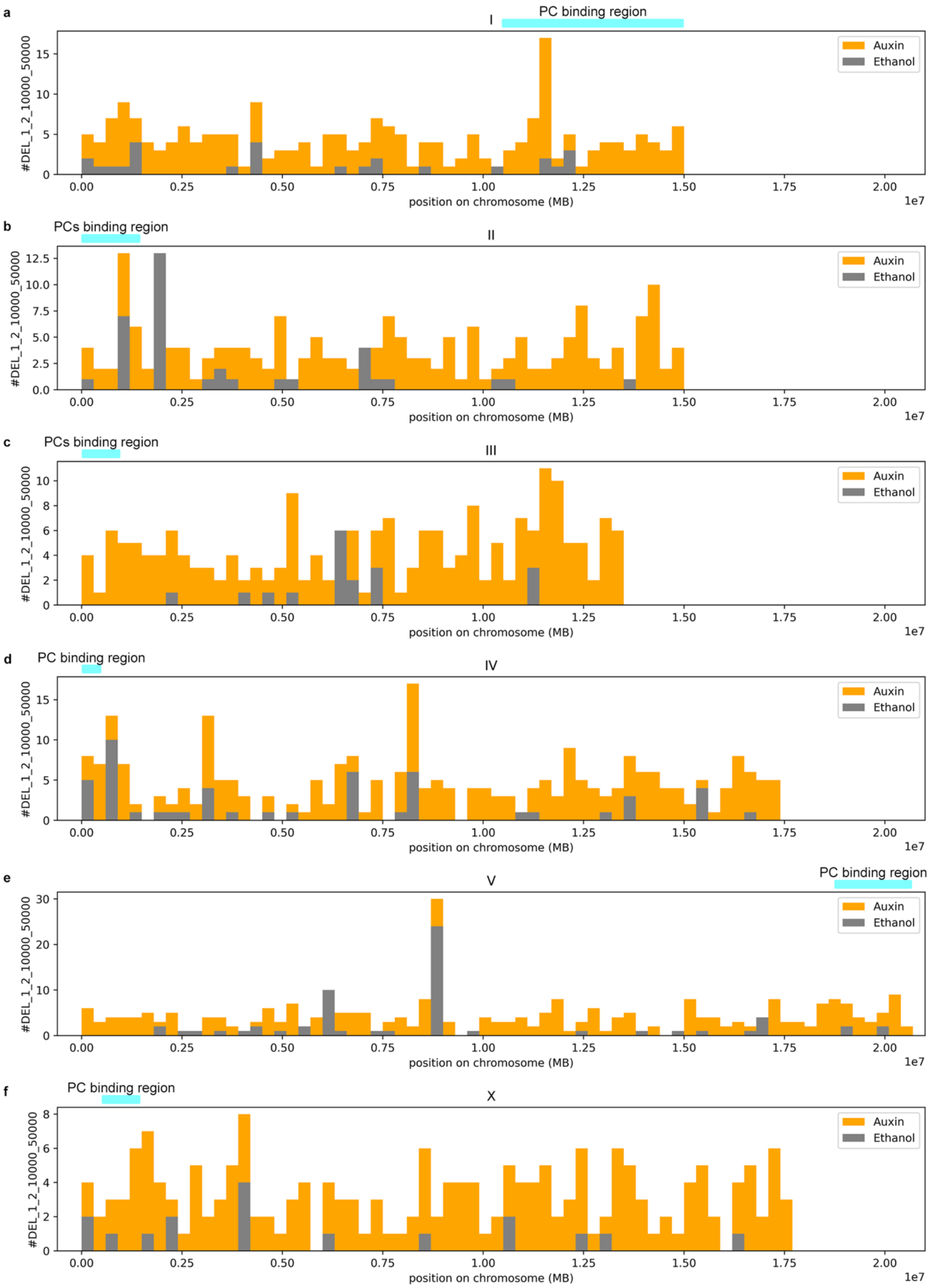

Supplementary Fig. 10. Number of DELs of size 10,000–50,000 bp supported by 1 and 2 reads along linkage groups I (**a**), II (**b**), III (**c**), IV (**d**), V (**e**), and X (**f**) in samples from ethanol-treated (gray) and auxin-treated (orange) worms. The PC binding region is indicated (cyan). PC = pairing center.

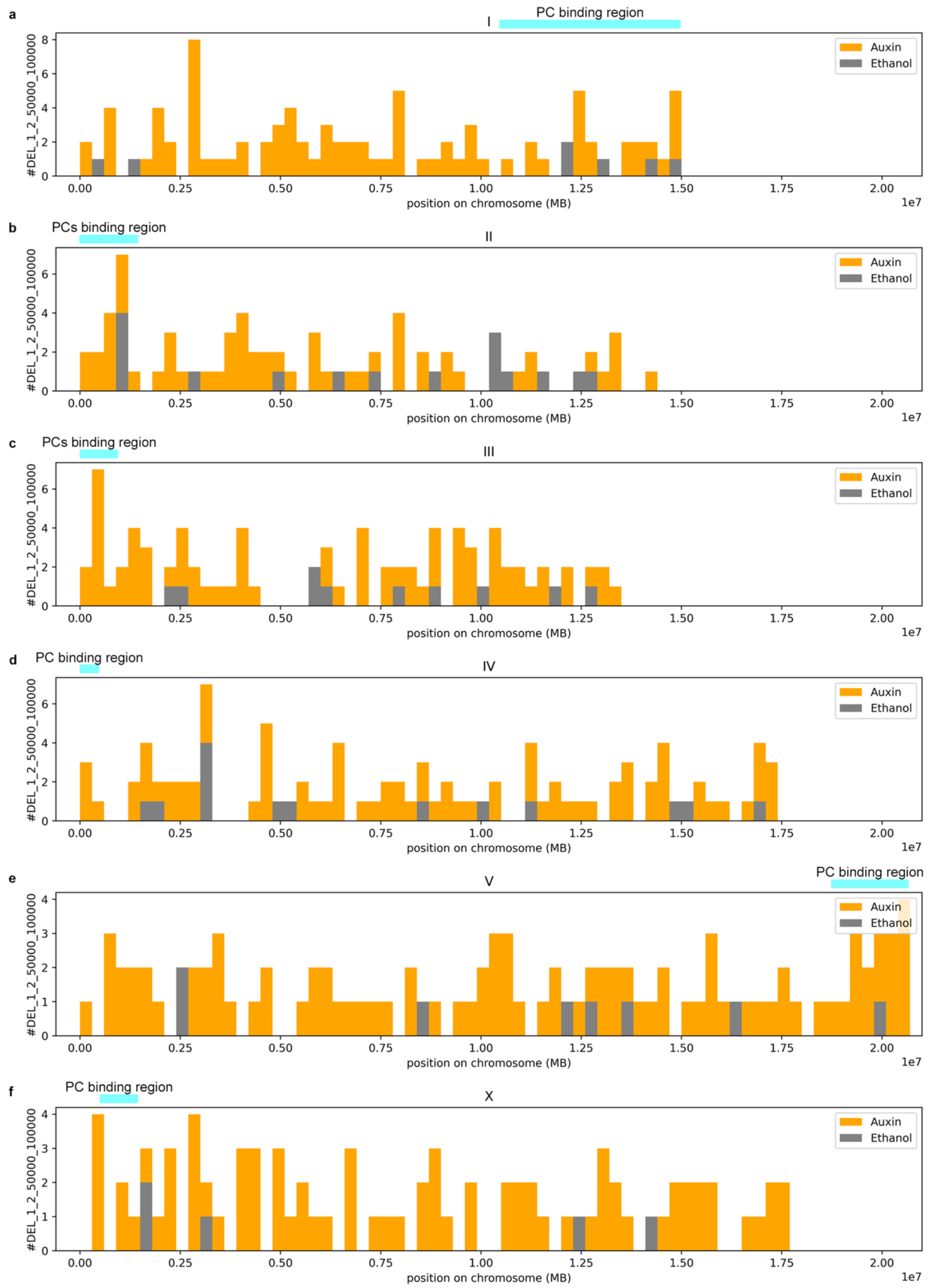

Supplementary Fig. 11. Number of DELs of size 50,000–100,000 bp supported by 1 and 2 reads along linkage group I (**a**), II (**b**), III (**c**), IV (**d**), V (**e**), and X (**f**) in samples from ethanol-treated (gray) and auxin-treated (orange) worms. The PC binding region is indicated (cyan). PC = pairing center.

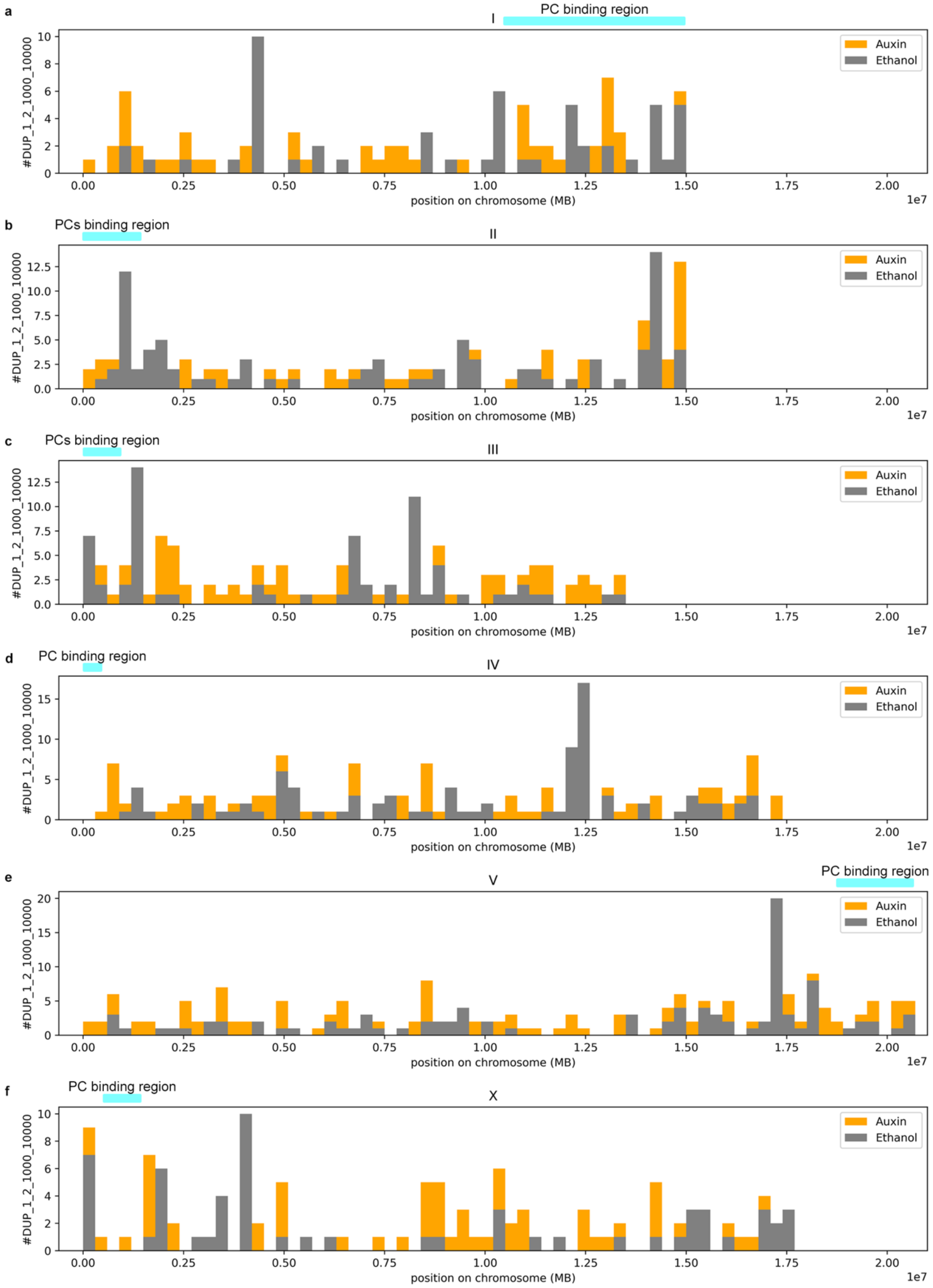

Supplementary Figs 12–14. Distribution of duplications along *C. elegans* chromosomes (bin size 300,000 bp)

Supplementary Fig. 12. Number of DUPs of size 1,000–10,000 bp supported by 1 and 2 reads along linkage groups I (**a**), II (**b**), III (**c**), IV (**d**), V (**e**), and X (**f**) in samples from ethanol-treated (gray) and auxin-treated (orange) worms. The PC binding region is indicated (cyan). PC = pairing center.

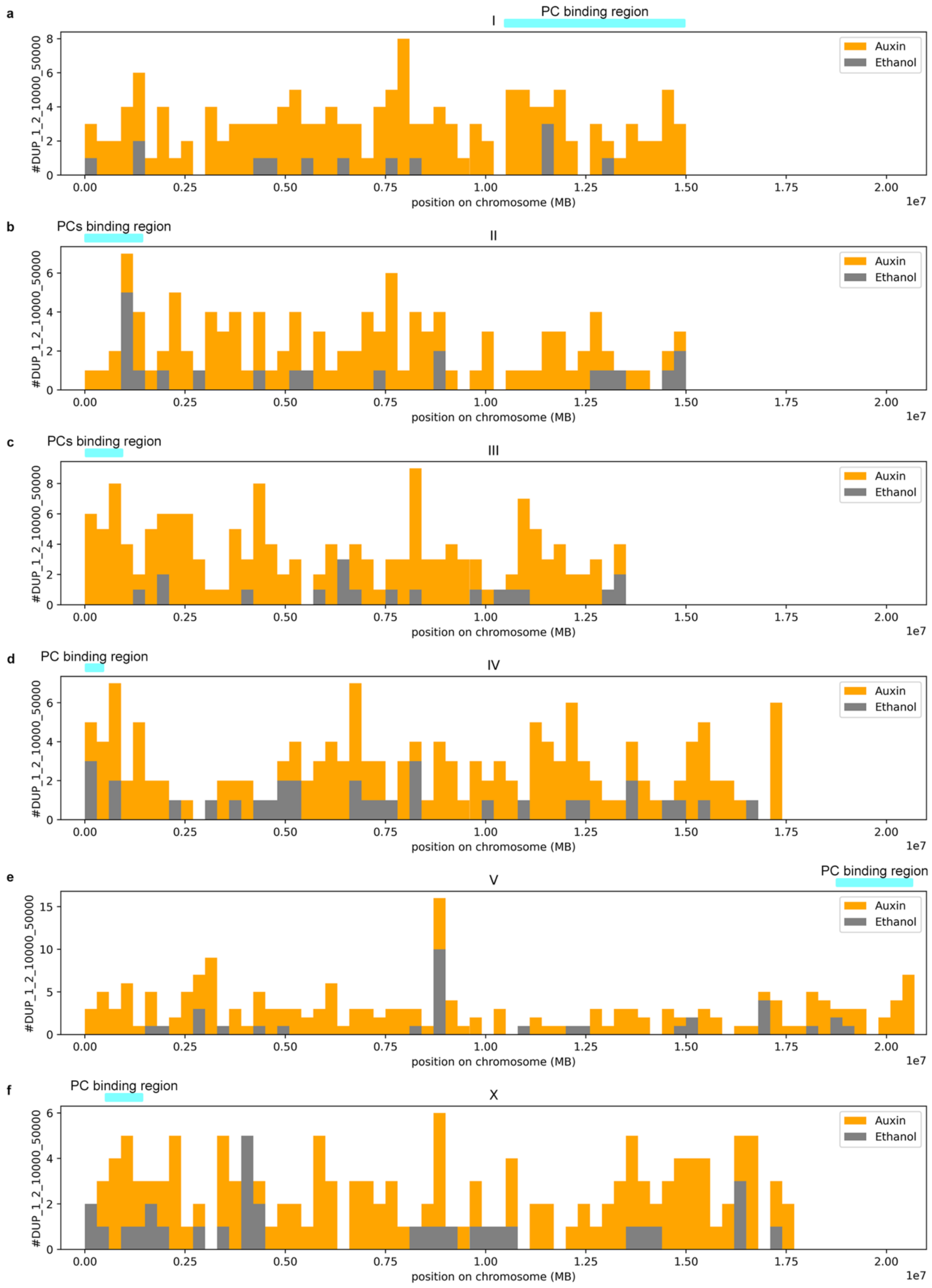

Supplementary Fig. 13. Number of DUPs of size 10,000–50,000 bp supported by 1 and 2 reads along linkage groups I (**a**), II (**b**), III (**c**), IV (**d**), V (**e**), and X (**f**) in samples from ethanol-treated (gray) and auxin-treated (orange) worms. The PC binding region is indicated (cyan). PC = pairing center.

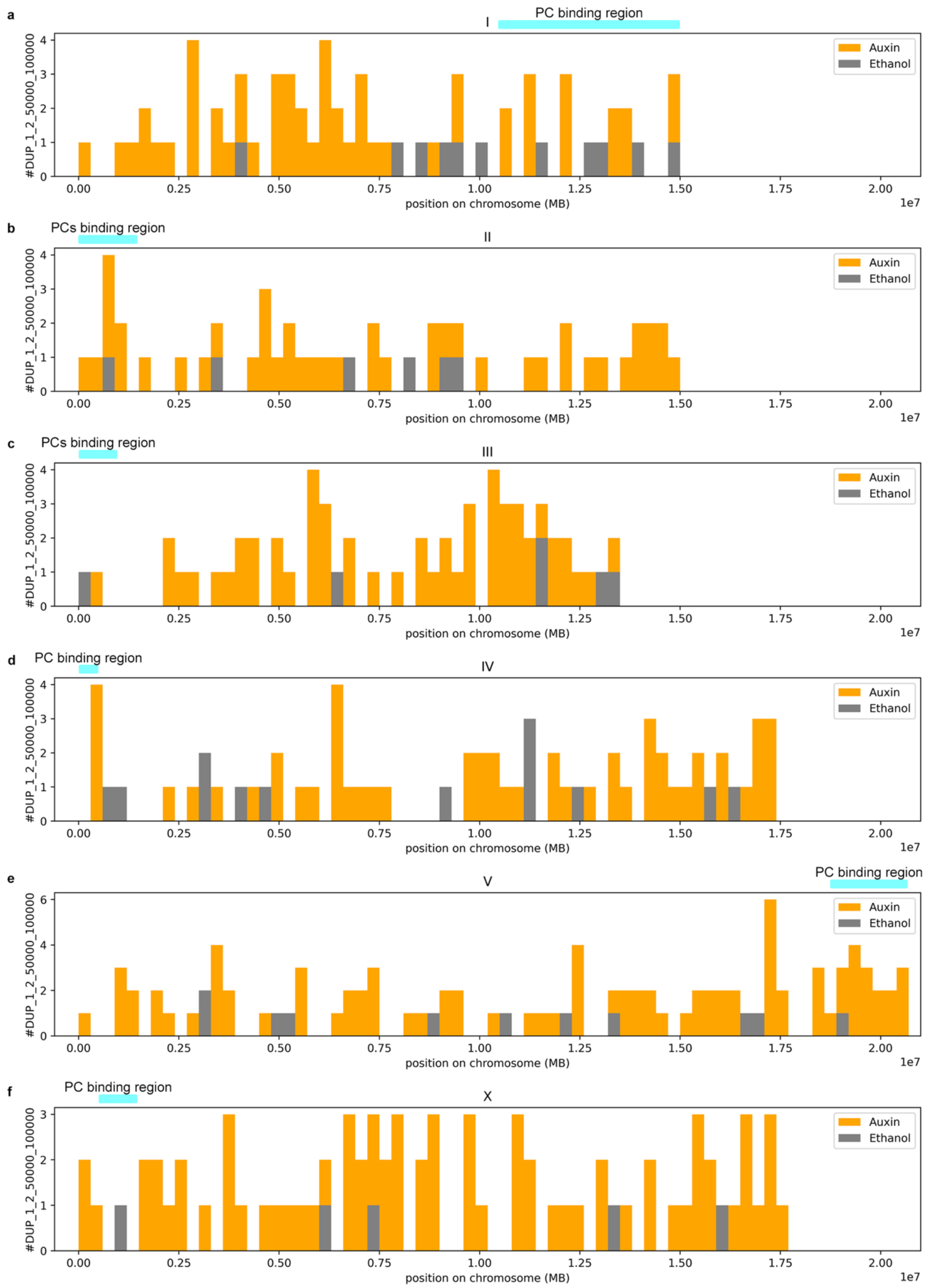

Supplementary Fig. 14. Number of DUPs of size 50,000–100,000 bp supported by 1 and 2 reads along linkage groups I (**a**), II (**b**), III (**c**), IV (**d**), V (**e**), and X (**f**) in samples from ethanol-treated (gray) and auxin-treated (orange) worms. The PC binding region is indicated (cyan). PC = pairing center.

Supplementary Figs 15–17. Distribution of insertions along *C. elegans* chromosomes (bin size 300,000 bp)

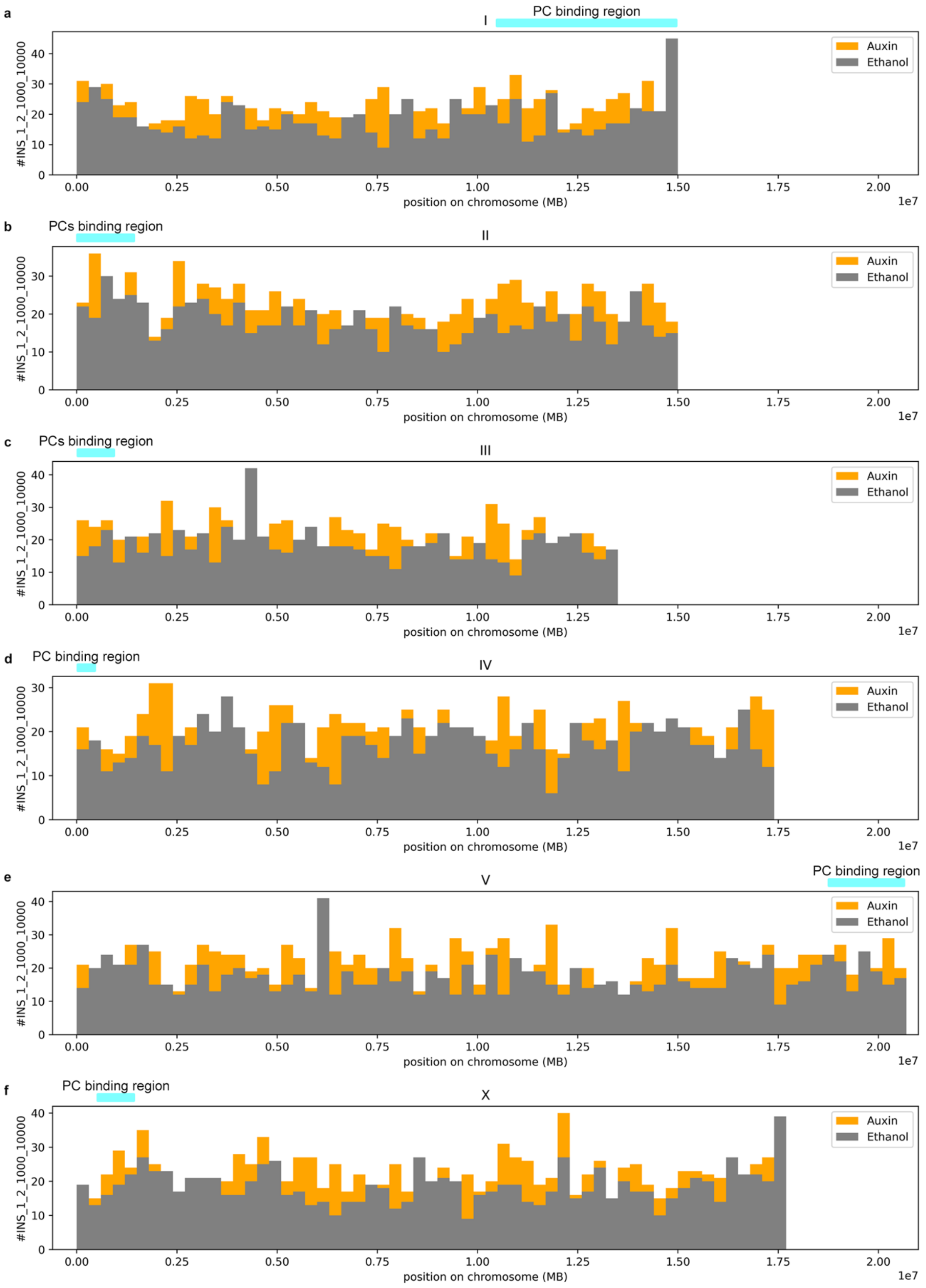

Supplementary Fig. 15. Number of INSs of size 1,000–10,000 bp supported by 1 and 2 reads along linkage groups I (**a**), II (**b**), III (**c**), IV (**d**), V (**e**), and X (**f**) in samples from ethanol-treated (gray) and auxin-treated (orange) worms. The PC binding region is indicated (cyan). PC = pairing center.

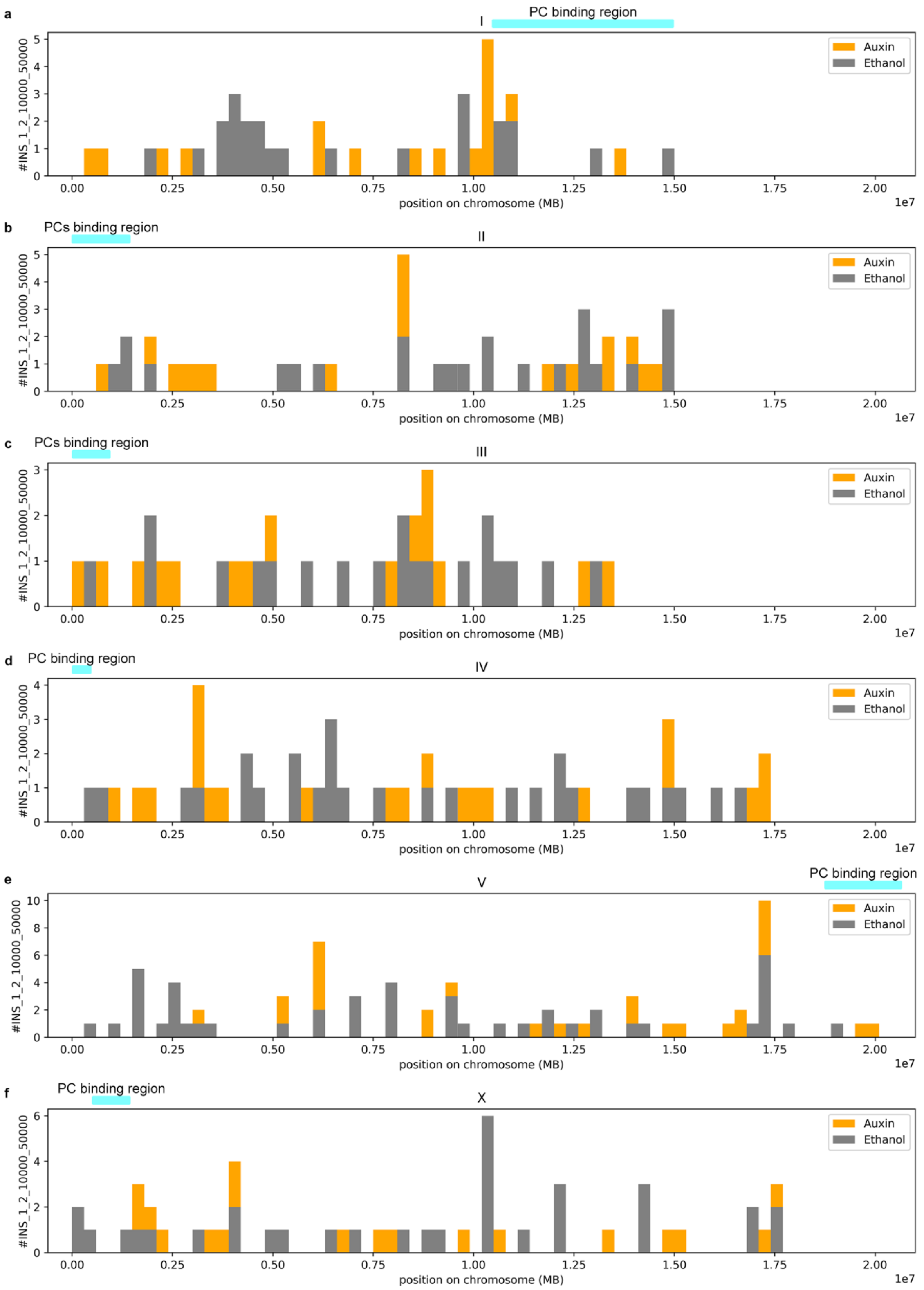

Supplementary Fig. 16. Number of INSs of size 10,000–50,000 bp supported by 1 and 2 reads along linkage groups I (**a**), II (**b**), III (**c**), IV (**d**), V (**e**), and X (**f**) in samples from ethanol-treated (gray) and auxin-treated (orange) worms. The PC binding region is indicated (cyan). PC = pairing center.

.

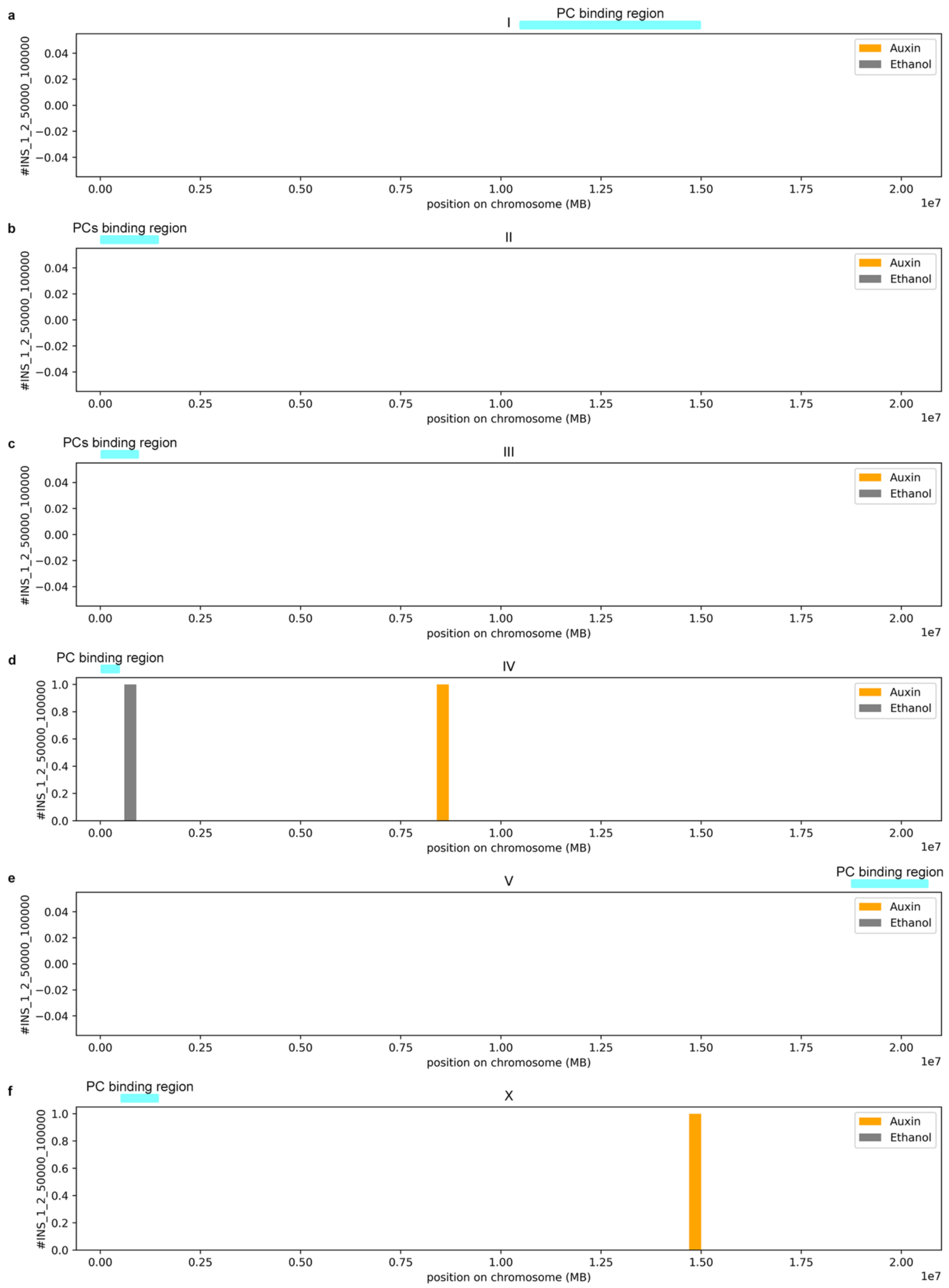

Supplementary Fig. 17. Number of INSs of size 50,000–100,000 bp supported by 1 and 2 reads along linkage groups I (**a**), II (**b**), III (**c**), IV (**d**), V (**e**), and X (**f**) in samples from ethanol-treated (gray) and auxin-treated (orange) worms. The PC binding region is indicated (cyan). PC = pairing center.

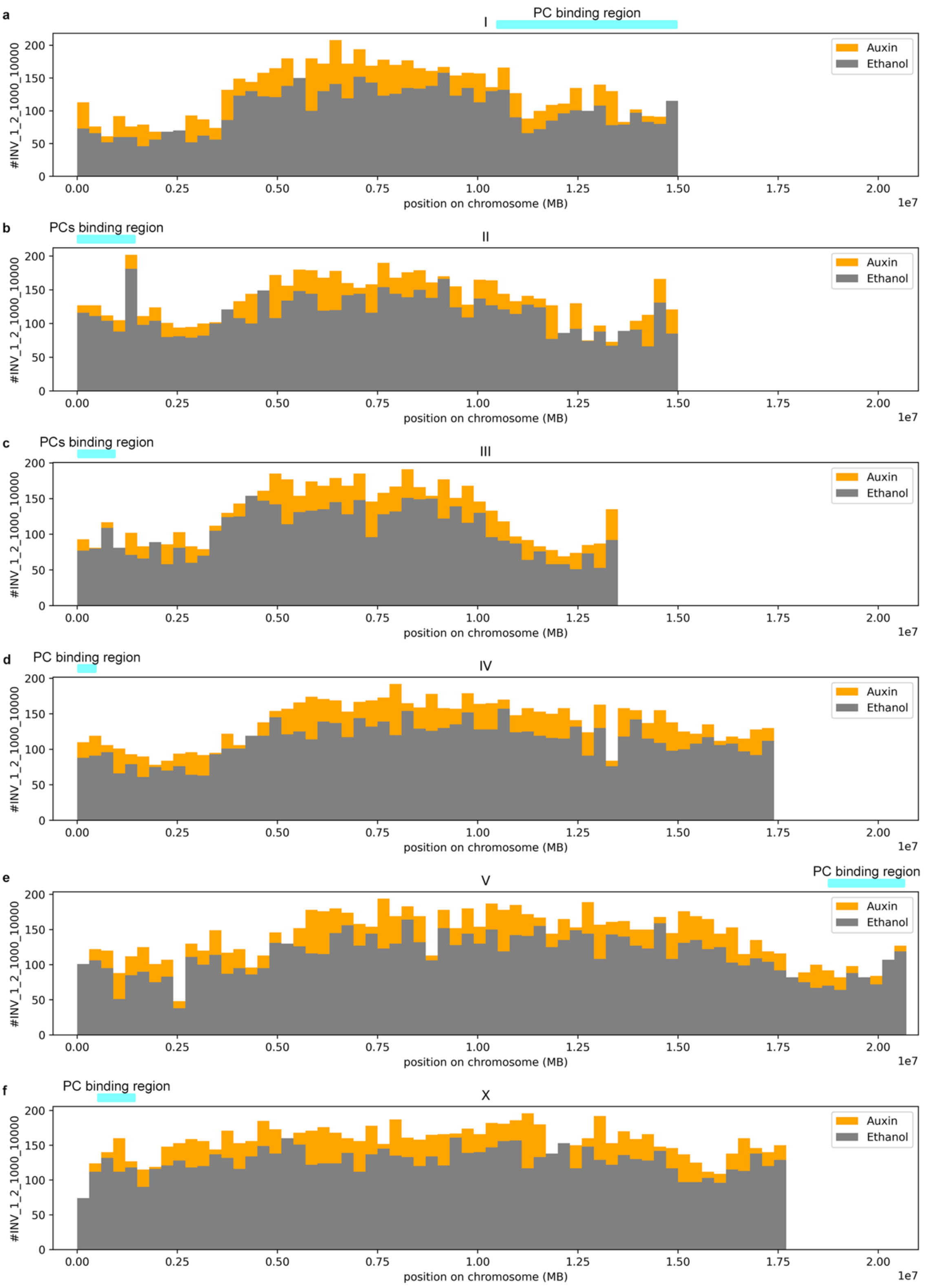

Supplementary Figs 18–20. Distribution of inversions along *C. elegans* chromosomes (bin size 300,000 bp)

Supplementary Fig. 18. Number of INVs of size 1,000–10,000 bp supported by 1 and 2 reads along linkage groups I (**a**), II (**b**), III (**c**), IV (**d**), V (**e**), and X (**f**) in samples from ethanol-treated (gray) and auxin-treated (orange) worms. The PC binding region is indicated (cyan). PC = pairing center.

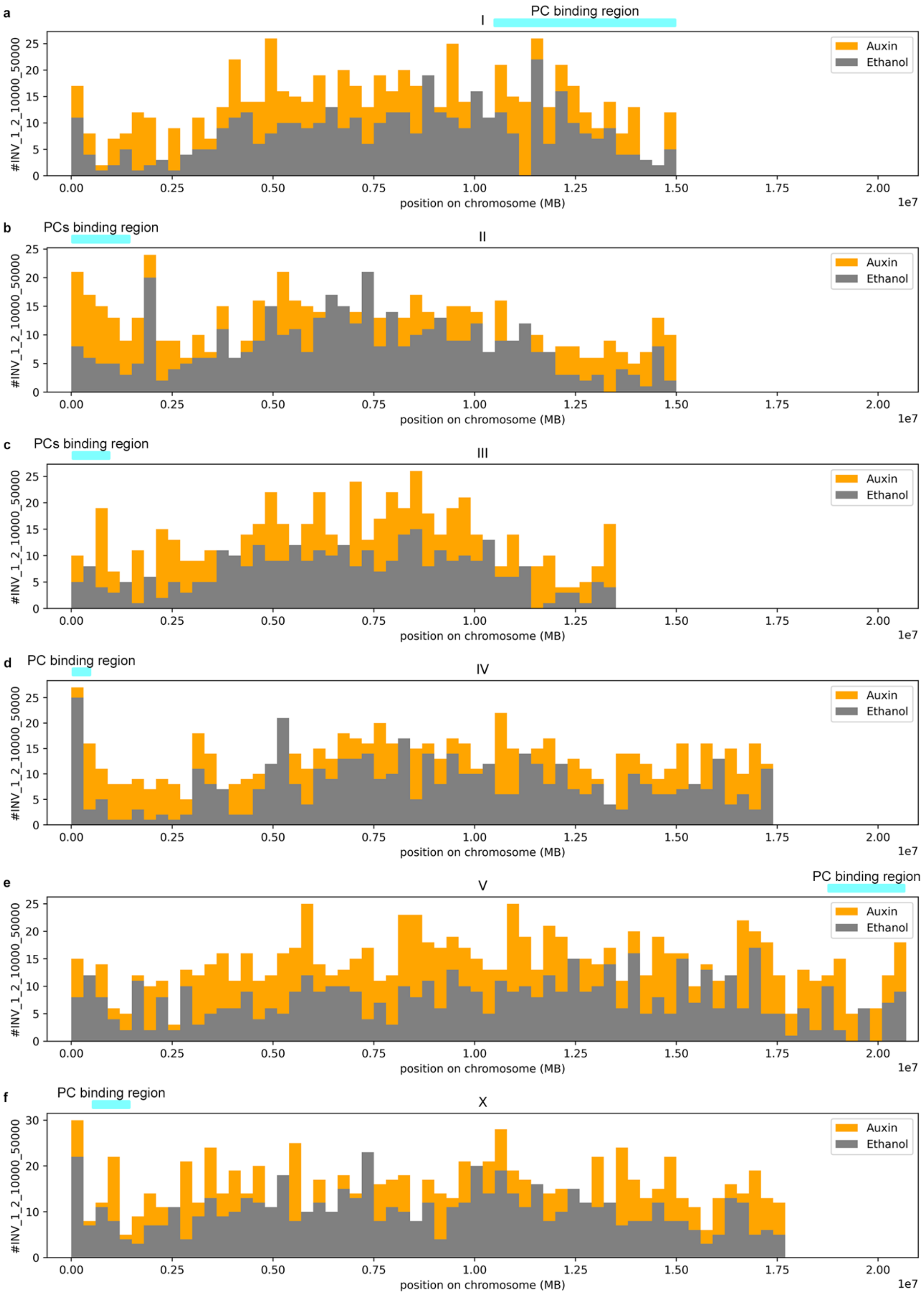

Supplementary Fig. 19. Number of INVs of size 10,000–50,000 bp supported by 1 and 2 reads along linkage groups I (**a**), II (**b**), III (**c**), IV (**d**), V (**e**), and X (**f**) in worms from ethanol-treated (gray) and auxin-treated (orange) worms. The PC binding region is indicated (cyan). PC = pairing center.

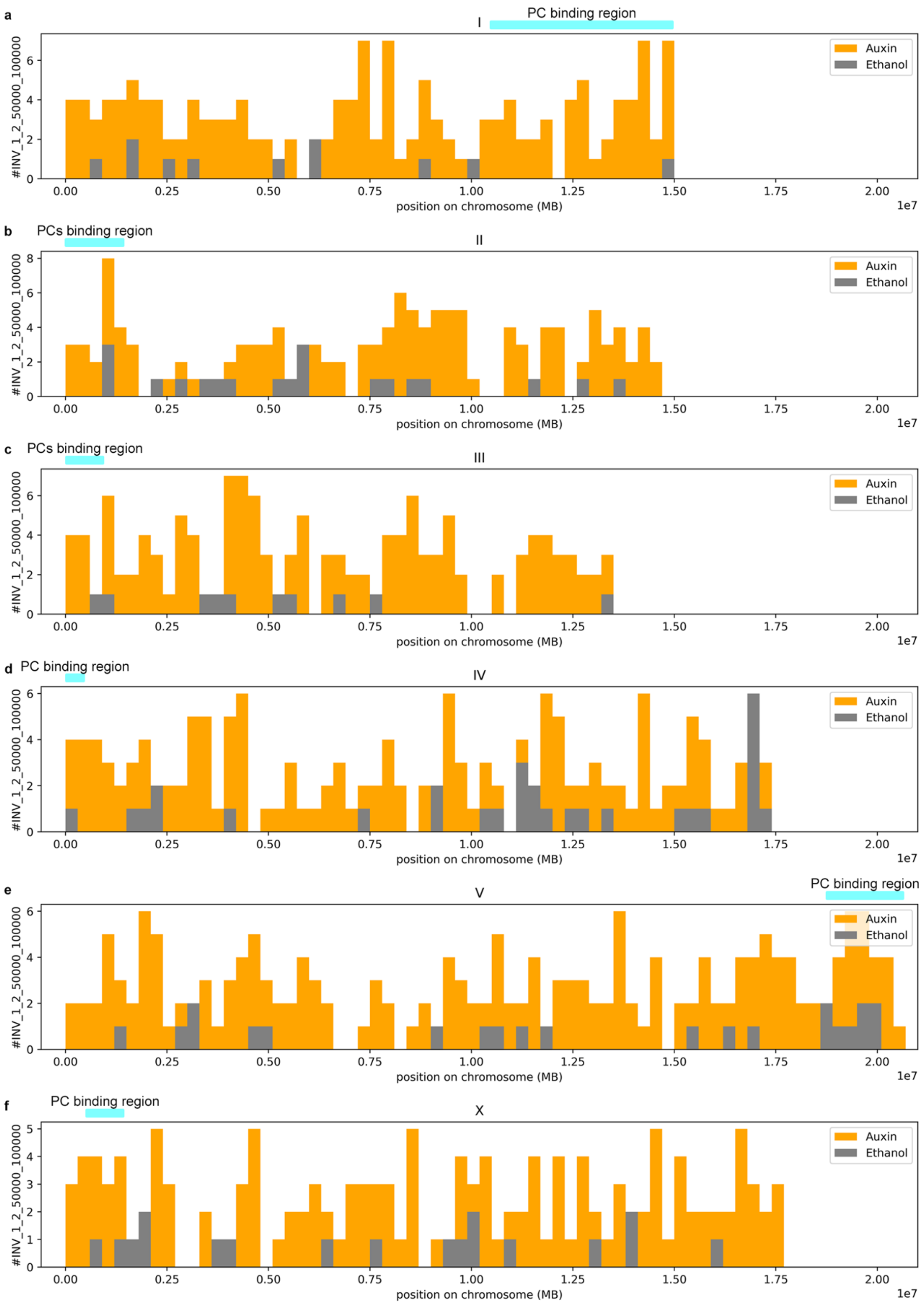

Supplementary Fig. 20. Number of INVs of size 50,000–100,000 bp supported by 1 and 2 reads along linkage groups I (**a**), II (**b**), III (**c**), IV (**d**), V (**e**), and X (**f**) in ethanol-treated (gray) and auxin-treated (orange) worms. The PC binding region is indicated (cyan). PC = pairing center.

Supplementary Methods

Line profile analysis

Microscopy images were acquired as previously described [1], with some modifications. Using ImageJ, a line of 35 pixels in width (covering the diameter of a mitotic or meiotic nucleus) was created to measure the anti-HA or anti-FLAG antibody signal and added to the region of interest (ROI) manager. At least 25 nuclei from the progenitor zone and 25 after meiotic entry were processed for each condition (ethanol or auxin treatment). After collection, line profiles were resampled with R software using the longest track as reference, averaged and plotted using GraphPad Prism 10.4.0 for Mac. HA = hemagglutinin

Quantification of migration rate, synaptonemal complex assembly, chromosome pairing, and GFP::MSH-5 foci

To determine the migration rate, SC assembly and chromosome pairing, germlines were divided into seven equal zones. In each zone, EdU-positive nuclei, nuclei with fully assembled SCs (fully colocalized HTP-3 and SYP-1 markers), and nuclei with paired chromosomes (one signal for the 5S locus) were counted. These data were normalized against the total number of nuclei per zone.

Owing to lack of a clear transition zone upon VRK-1 depletion, we counted GFP::MSH-5 foci numbers per nucleus in 30 cell rows from the end of late pachynema until the transition zone, divided in three zones: rows 1–10, 11–20 and 21–30, numbered from diplonema onwards.

Quantification of chromatin clustering and length of the mitotic zone

To quantify nuclei with clustered chromatin, we used anti-PiSer8 SUN-1 antibody staining to mark meiotic entry and DAPI to visualize chromatin (≥ 50% of nuclei in one cell row were positive for PiSer8 SUN-1). The number of nuclei with clustered chromatin was normalized against the total number of nuclei in this zone. Graphs show the percentage of nuclei with clustered chromatin for each genotype.

To quantify mitotic length (Supplementary Fig. 2g), we counted the cell rows from the distal tip to meiotic entry (positive for PiSer8 SUN-1) and normalized this number to the total number of cell rows (from distal tip to diplonema). Graphs show the percentage of the mitotic length to germline length for each genotype.

3D analysis of diakinesis

Stack pictures acquired with a DeltaVision microscope (100× objective) were processed using Napari software [2]. From the DAPI channel, ROIs were created using the PartSeg toolbox [3] with the following parameters: filter, Gaussian, stack, radius 1; threshold, moments; histogram bins, 20; and minimum

size, 200. Detected ROIs were saved as a tiff file. To create a LMN-1 mask, the PartSeg toolbox was used with the following parameters: filter, Gaussian, stack, radius 1; threshold, moments; histogram bins, 25; and minimum size, 70,000. The mask was saved as a tiff file. To create the SUN-1(S8Pi) mask, the PartSeg toolbox was used with the following parameters: Filter: Bilateral, stack, radius 1, Threshold: Otsu, Histogram bins: 250, Minimum size:60,000 and the mask saved as tiff file as well. To measure the distances between the ROIs detected from the DAPI channel and LMN-1 or SUN-1 (S8Pi) mask, images were opened in FIJI [4] and calibrated (x =y = 0.0643 nm, z = 0.2 nm). Before the addition of the LMN-1 or SUN-1(S8Pi) to the 3D analysis toolbox [5], the LMN-1 or SUN-1(S8Pi) mask were expanded twice and the holes filled to ensure the correct delimitation of its periphery. Distances between LMN-1 or SUN-1 (S8Pi) and ROIs were then measured. Data were plotted and statistical analysis was done using GraphPad Prism 10.4.0 for Mac.

Oligopainting acquisition and quantification

Image stacks were acquired using a Zeiss LSM900 confocal microscope with Airyscan 2 detection and processed for three-dimensional (3D) analysis. Images were captured using a 63×/1.4 Oil differential interference contrast (DIC) objective, with excitation at 405, 488, 561, and 640 nm to detect DAPI staining and probes staining linkage groups (LG) I, II, and III, respectively. Following data acquisition, Airyscan processing was performed using Zeiss Zen software to enhance resolution. From the resulting images, nuclei were manually extracted, split into separate channels in FIJI [4], and reopened in Napari [2] for segmentation. ROIs were isolated using PartSeg toolbox [3] with the following parameters: for LGI and LGII – filter, Gaussian, stack, radius 1; threshold: Li; histogram bins, 15; and minimum size, 5000; and for LGIII – filter, Gaussian, stack, radius 1; threshold, Otsu; histogram bins: 125; and minimum size, 5000. Detected ROIs were saved as a TIFF file.

Colocalization analysis of fluorescent channel masks was performed using custom Python scripts (available upon request) with the NumPy, imageio, and pandas libraries. The script processes TIFF images corresponding to three fluorescent channels (green, red, and far red) for each sample, groups files by experimental condition and sample ID, and then loads and converts the images to binary masks. The total number of voxels per channel and pairwise voxel overlaps between channels are computed. Total voxel counts and colocalization statistics are then compiled and saved as a CSV file for subsequent analysis.

Oxford Nanopore Technology sequence analysis

Ethanol (wild type) and auxin (*vrk-1::AID*) treated samples were sequenced on the PromethION nanopore sequencer at the Vienna BioCenter Next Generation Sequencing facility. All obtained

sequencing reads have been uploaded on the NCBI public database and are available under the project PRJNA1304376 (https://www.ncbi.nlm.nih.gov/sra/PRJNA1304376).

After base calling using Dorado (https://github.com/nanoporetech/dorado), 9714973.0 reads were recovered for the wild type sample (read length, median ± SD: 4022.0 ± 8608.8) and 9069950.0 reads for the *vrk-1::AID::ha* sample (read length, median ± SD: 6342.0 ± 10731.5). As differences in the distribution of read lengths can influence the number of structural variants (SVs) called, we matched the read length distributions of the two libraries by binning the read lengths of the control sample into 100 equally sized bins and sampled the same number of reads from each bin in both samples (using the scripts provided on the GitHub page). We mapped the subsampled reads to the *C. elegans* genome retrieved from Ensembl (https://ftp.ensembl.org/pub/release-112/fasta/caenorhabditis_elegans/dna/, GenBank assembly ID: GCA_000002985.3, assembly name: WBcel235) using ngmlr (-x ont, [6]). The resulting Sequence Alignment Map (SAM) files were converted to the Binary Alignment Map (BAM) format, sorted, and indexed using samtools [7]. The BAM files were then used to call SVs in the wild type and *vrk-1::AID::ha* samples with both Sniffles [8] and cuteSV [9] and the two sets of results were compared. For cuteSV, we used the default parameters for ONT data (minimum quality, 20; maximum length, 100,000) with the --min_support 1 option. For Sniffles, we used the following options: --qc-output-all --mosaic --tandem-repeats celegans.trf.bed --threads 60 --output-rnames (the --qc-output-all option was included to allow the detection of low frequency variants). The tandem repeats file was generated using findTandemRepeats, which is provided by Pacific Biosciences as part of the pbsv package (https://github.com/PacificBiosciences/pbsv). The VCF files generated by Sniffles and cuteSV were parsed and analyzed using the Python scripts/Jupyter notebooks provided on the GitHub page (https://doi.org/10.5281/zenodo.17132536)[10].The pairing centers binding regions were indicated according to [11].

Analysis for presence of repeats at the SV sites

SV breakpoints were analyzed for the presence of short tandem repeats (STRs) with unit sizes of 1, 2, or 3 bp using STR-Finder (https://zenodo.org/records/17151593)[12]. The analysis located STRs within 20 bp upstream or downstream of each SV breakpoint using coordinates mapped to the *C. elegans* reference genome (GenBank assembly ID: GCA_000002985.3, assembly name: WBcel235). The repeat content at the flanking sites in *vrk-1::AID::ha* worms (ethanol and auxin treated), including genomic coordinates and the repeat length and type, are listed in Supplementary Data 1.

Reagents

Supplementary Table 3: Strains generated and used for this study

**Strain**[1]	**Genotype**	**Generated**
PHX2608	*vrk-1(syb2608[vrk-1::degron::ha])*	This study
CA1199	*ieSi38 [Psun-1 TIR-1::mRuby::sun-1 3'UTR, cb-unc-119(+)] IV*	[13]
UV310	*vrk-1(syb2608[vrk-1::degron::ha]); unc-119(ed3) III; ieSi38 [Psun-1 TIR-1::mRuby::sun-1 3'UTR, cb-unc-119(+)] IV*	This study
UV311	*baf-1(jf206(1xflag::baf-1)) III*	This study
UV312	*baf-1(jf206(1Xflag::baf-1)) III; vrk-1(syb2608 vrk-1::degron::ha)II; unc-119(ed3) III; ieSi38 [Psun-1 TIR-1::mRuby::sun-1 3'UTR, cb-unc-119(+)]*	This study
UV313	*vrk-1(syb2608 vrk-1::degron::ha) JfSi1[Psun-1::GFP cb-unc-119(+)] II; unc-119(ed3) III; ieSi38 [Psun-1 TIR-1::mRuby::sun-1 3'UTR, cb-unc-119(+)] IVsun-1(ok1282)V*	This study
UV314	*vrk-1(syb2608 vrk-1::degron::ha)II; unc-119(ed3) III; spo-11(ok79) ieSi38 [Psun-1 TIR-1::mRuby::sun-1 3'UTR, cb-unc-119(+)] IV/nT1*	This study
UV315	*vrk-1(syb2608 vrk-1::degron::ha)II; unc-119(ed3) III; ieSi38 [Psun-1 TIR-1::mRuby::sun-1 3'UTR, cb-unc-119(+)] GFP::msh-5 IV*	This study
UV316	*vrk-1(syb2608 vrk-1::degron::ha)II; unc-119(ed3) III; ieSi38 [Psun-1 TIR-1::mRuby::sun-1 3'UTR, cb-unc-119(+)] msh-5(me23) IV/nT1 [unc-?(n754) let-?] (IV;V)*	This study
UV317	*mus-81(tm1937) I;vrk-1(syb2608 vrk-1::degron::ha)II; unc-119(ed3) III; ieSi38 [Psun-1 TIR-1::mRuby::sun-1 3'UTR, cb-unc-119(+)] IV*	This study
UV318	*polq-1(tm2026) III, vrk-1(syb2608 vrk-1::degron::ha)II; unc-119(ed3) III; ieSi38 [Psun-1 TIR-1::mRuby::sun-1 3'UTR, cb-unc-119(+)] IV*	This study
UV319	*vrk-1(syb2608 vrk-1::degron::ha)II; lig-4(ok716) III; unc-119(ed3) III; ieSi38 [Psun-1 TIR-1::mRuby::sun-1 3'UTR, cb-unc-119(+)] IV*	This study
UV320	*vrk-1(syb2608 vrk-1::degron::ha)II; cku-70 (tm1524) III; unc-119(ed3) III; ieSi38 [Psun-1 TIR-1::mRuby::sun-1 3'UTR, cb-unc-119(+)] IV*	This study
UV321	*dtn-1::FLAG::GFP vrk-1(syb2608[vrk-1::degron::HA]) (LGII); unc-119(ed3)*	This study

[1] Italics designates the gene and allele names.

	(LGIII); ieSi38 [Psun-1 TIR-1::mRuby::sun-1 3'UTR, cb-unc-119(+)] (LGIV)	
UV322	*baf-1 (jf215(1Xflag::baf-1(T3A)))*	This study
UV323	*baf-1 (jf216(1Xflag::baf-1(S4A)))III; qC1 [dpy-19(e1259) glp-1(q339) qIs26] III/eT1 (III;V)*	This study
UV324	*sun-1(jfSi1[Psun-1::GFP cb-unc-119(+)]) (LGII); baf-1(jf216[1xflag::baf-1(S4A)]) (LGIII) qC1 [dpy-19(e1259) glp-1(q339) qIs26] III/eT1 (III;V); sun-1(ok1282) (LGV)*	This study
TG2228	*polq-1(tm2026) III*	[14]
PHX2016	*dtn-1::flag::gfp II*	[15]
RB873	*lig-4(ok716) III*	*C. elegans* deletion consortium
FX1524	*cku-70 (tm1524) III*	National Bioresource Project at the Tokyo Women's Medical University School of Medicin

Supplementary Table 4: crRNAs and repair templates designed for this study

Gene	crRNA (20bp + NGG, 5'–3')	Repair template (5'–3')
flag:baf-1	ATGCTTAACAGA AGTCGACATGG	TCTCCGACGAACTCACGATGCTTAACAGAAGT CGATCCACTTCCTCCCTTGTCATCGTCATCCTTGTAATCCATGGTTT CTGAAACACAAAATAATTACATTCTTGAATTT
flag::baf-1^{T3A}	GTTAAGCATCGT GAGTTCGTCGG	AAACCATGGATTACAAGGATGACGATGACAAGGGAGGAAGTGG ATCGGCTTCTGTTAAACATCGCGAATTTGTCGGAGAGCCAATGGG CGACAAAGAAGTCACATGCATCGCCGGGATCGGGCCAAC
flag::baf-1^{S4A}	GTTAAGCATCGT GAGTTCGTCGG	AAACCATGGATTACAAGGATGACGATGACAAGGGAGGAAGTGG ATCGACTGCTGTTAAACATCGCGAATTTGTCGGAGAGCCAATGGG CGACAAAGAAGTCACATGCATCGCCGGGATCGGGCCAAC

Supplementary Table 5: Primers designed for this study

Gene	Forward 5'–3'	Reverse 5'–3'
vrk-1::degron::ha	TGTTAACTAGGTAAATATAGC	CTGAAACCAATCAGGTCAGC
flag::baf-1	TTCTCAGCCCTTGCATCACC	GAACTGATCTGCCCACTCGT
flag::baf-1^{T3A}	As for *flag::baf-1* followed by NruI restriction	
flag::baf-1^{S4A}	As for *flag::baf-1* followed by NruI restriction	

All crRNAs, single strand oligonucleotides were ordered by IDT™ and sequencing primers from Microsynth.

Supplementary Table 6: Antibodies used in this study

Primary antibodies	Antibody source, dilution used
BAF-1 Ser4Pi (rabbit)	This study, 1:200
FLAG (rabbit)	Sigma-Aldrich, cat. number F7425, 1:300
FLAG (mouse)	Sigma-Aldrich, cat. number F3165, precleaned against N2 and used 1:250

GAPDH	Thermofischer Scientific, cat. number AM4300, 1:5000
HA (rabbit)	Sigma-Aldrich, cat. number H6908, precleaned against N2 and used 1:100
H3	Abcam, cat. number ab1791, 1:25000
HTP-3 (guinea pig)	From Yumi Kim, 1:500
LMN-1 (guinea pig)	From Georg Krohne, Wurzbürg 1:2000
SUN-1 Se8Pi (guinea pig)	Generated in our lab, 1:700
SYP-1 (rabbit)	From Nicola Silva, 1:1000
PLK-2	From Rueyling Kin, 1:500
ZHP-3	From Nheedi Bhalla, 1:500
**Secondary antibodies**	**Antibody source, dilution used**
Mouse	Thermofischer Scentific, cat. number A11032 (1:500), A11001 (1:500), Biotium, cat. number 20014 (1:200), Cell Signalling, cat. number 7076 (1:2500)
Guinea pig	Thermofischer Scentific, cat. number A11073 (1:500), A21450 (1:200)
Rabbit	Thermofischer Scientific, cat. number A11036 (1:500), A11034 (1:500), A21245 (1:200), 31460 (1:25000)

Supplementary References

1 Baudrimont, A. *et al.* Release of CHK-2 from PPM-1.D anchorage schedules meiotic entry. *Sci Adv* **8**, eabl8861, doi:10.1126/sciadv.abl8861 (2022).

2 Sofroniew, N. *et al.* napari: a multi-dimensional image viewer for Python. doi:10.5281/zenodo.3555620 (2019).

3 Bokota, G. *et al.* PartSeg: a tool for quantitative feature extraction from 3D microscopy images for dummies. *BMC Bioinformatics* **22**, 72, doi:10.1186/s12859-021-03984-1 (2021).

4 Schindelin, J. *et al.* Fiji: an open-source platform for biological-image analysis. *Nature Methods* **9**, 676-682, doi:10.1038/nmeth.2019 (2012).

5 Ollion, J., Cochennec, J., Loll, F., Escudé, C. & Boudier, T. TANGO: a generic tool for high-throughput 3D image analysis for studying nuclear organization. *Bioinformatics* **29**, 1840-1841, doi:10.1093/bioinformatics/btt276 (2013).

6 Sedlazeck, F. J. *et al.* Accurate detection of complex structural variations using single-molecule sequencing. *Nat Methods* **15**, 461-468, doi:10.1038/s41592-018-0001-7 (2018).

7 Li, H. *et al.* The Sequence Alignment/Map format and SAMtools. *Bioinformatics* **25**, 2078-2079, doi:10.1093/bioinformatics/btp352 (2009).

8 Smolka, M. *et al.* Detection of mosaic and population-level structural variants with Sniffles2. *Nat Biotechnol* **42**, 1571-1580, doi:10.1038/s41587-023-02024-y (2024).

9 Jiang, T. *et al.* Long-read-based human genomic structural variation detection with cuteSV. *Genome Biol* **21**, 189, doi:10.1186/s13059-020-02107-y (2020).

10 Marwan Elkrewi, R. K., Beatriz Vicoso. BAF-1–VRK-1 mediated release of meiotic chromosomes from the nuclear periphery is important for genome integrity. doi:https://doi.org/10.5281/zenodo.17132536 (2025).
11 Phillips, C. M. *et al.* Identification of chromosome sequence motifs that mediate meiotic pairing and synapsis in C. elegans. *Nat Cell Biol* **11**, 934-942, doi:10.1038/ncb1904 (2009).
12 Shehab Moukbel Ali Aldawla, I. T.-B. BAF-1–VRK-1 mediated release of meiotic chromosomes from the nuclear periphery is important for genome integrity. doi:https://doi.org/10.5281/zenodo.17151593 (2025).
13 Zhang, L., Ward, J. D., Cheng, Z. & Dernburg, A. F. The auxin-inducible degradation (AID) system enables versatile conditional protein depletion in C. elegans. *Development* **142**, 4374-4384, doi:10.1242/dev.129635 (2015).
14 Muzzini, D. M., Plevani, P., Boulton, S. J., Cassata, G. & Marini, F. Caenorhabditis elegans POLQ-1 and HEL-308 function in two distinct DNA interstrand cross-link repair pathways. *DNA Repair (Amst)* **7**, 941-950, doi:10.1016/j.dnarep.2008.03.021 (2008).
15 Yamamoto, I., Zhang, K., Zhang, J., Vorontsov, E. & Shibuya, H. Telomeric double-strand DNA-binding proteins DTN-1 and DTN-2 ensure germline immortality in Caenorhabditis elegans. *Elife* **10**, doi:10.7554/eLife.64104 (2021).
