# Supplementary material for: BAF-1–VRK-1 mediated release of meiotic chromosomes from the nuclear periphery is important for genome integrity
Source: Nat Commun. 2025 Nov 25;16:10446. doi: 10.1038/s41467-025-65420-9 (PMC12647756; doi:10.1038/s41467-025-65420-9)
Supplement: Supplementary file 2 — Description of Additional Supplementary Files [file 41467_2025_65420_MOESM2_ESM.pdf]

Description of Additional Supplementary Files

Supplementary Data 1: Repeat content at the flanking sites of the genome structural variants in *vrk-1::AID::ha* worms (ethanol and auxin treated), including genomic coordinates and the repeat length and type.
